# Interactions between Gepotidacin and *Escherichia coli* Gyrase and Topoisomerase IV: Genetic and Biochemical Evidence for Well-Balanced Dual-Targeting

**DOI:** 10.1021/acsinfecdis.3c00346

**Published:** 2024-03-05

**Authors:** Alexandria A. Oviatt, Elizabeth G. Gibson, Jianzhong Huang, Karen Mattern, Keir C. Neuman, Pan F. Chan, Neil Osheroff

**Affiliations:** † Department of Biochemistry, Vanderbilt University School of Medicine, Nashville, Tennessee 37232, United States; ‡ Department of Pharmacology, Vanderbilt University School of Medicine, Nashville, Tennessee 37232, United States; § Department of Medicine (Hematology/Oncology), Vanderbilt University School of Medicine, Nashville, Tennessee 37232, United States; ∥ Infectious Diseases Research Unit, GlaxoSmithKline, Collegeville, Pennsylvania 19426, United States; ⊥ Laboratory of Single Molecule Biophysics, National Heart, Lung, and Blood Institute, National Institutes of Health, Bethesda, Maryland 20982, United States; # VA Tennessee Valley Healthcare System, Nashville, Tennessee 37212, United States

**Keywords:** gyrase, topoisomerase IV, gepotidacin, triazaacenaphthylene, DNA cleavage, DNA supercoiling/decatenation

## Abstract

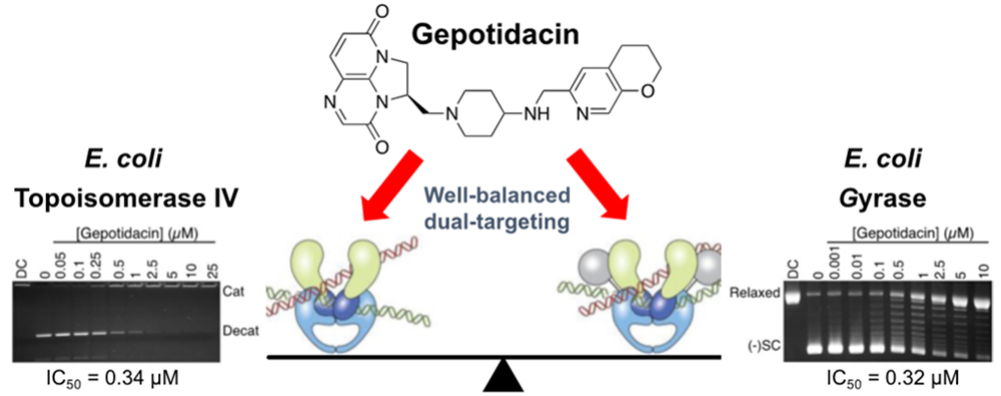

Antimicrobial resistance is a global threat to human health. Therefore, efforts have been made to develop new antibacterial agents that address this critical medical issue. Gepotidacin is a novel, bactericidal, first-in-class triazaacenaphthylene antibacterial in clinical development. Recently, phase III clinical trials for gepotidacin treatment of uncomplicated urinary tract infections caused by uropathogens, including *Escherichia coli*, were stopped for demonstrated efficacy. Because of the clinical promise of gepotidacin, it is important to understand how the compound interacts with its cellular targets, gyrase and topoisomerase IV, from *E. coli*. Consequently, we determined how gyrase and topoisomerase IV mutations in amino acid residues that are involved in gepotidacin interactions affect the susceptibility of *E. coli* cells to the compound and characterized the effects of gepotidacin on the activities of purified wild-type and mutant gyrase and topoisomerase IV. Gepotidacin displayed well-balanced dual-targeting of gyrase and topoisomerase IV in *E. coli* cells, which was reflected in a similar inhibition of the catalytic activities of these enzymes by the compound. Gepotidacin induced gyrase/topoisomerase IV-mediated single-stranded, but not double-stranded, DNA breaks. Mutations in GyrA and ParC amino acid residues that interact with gepotidacin altered the activity of the compound against the enzymes and, when present in both gyrase and topoisomerase IV, reduced the antibacterial activity of gepotidacin against this mutant strain. Our studies provide insights regarding the well-balanced dual-targeting of gyrase and topoisomerase IV by gepotidacin in *E. coli*.

Antimicrobial resistance is a growing medical concern. Worldwide, over a million people died from resistant bacterial infections in 2019.^1^ If nothing is done to address this critical issue, it is estimated that the death toll may rise to 10 million per year by 2050.^2^

Fluoroquinolones, which target gyrase and topoisomerase IV, are listed by the World Health Organization as one of the five “highest priority critically important antimicrobials” currently in clinical use.^3^ Unfortunately, the clinical utility of this drug class is being diminished by the rise in mutations in gyrase and/or topoisomerase IV (i.e., target-mediated resistance).^4−10^ To address the critical issue of drug resistance, new antibacterial drug classes are being developed.^8,9,11−14^ Of these, the antibacterial that has progressed the furthest in clinical trials is gepotidacin,^15−22^ a novel first-in-class triazaacenaphthylene bacterial topoisomerase inhibitor (Figure 1).

**Figure 1:**
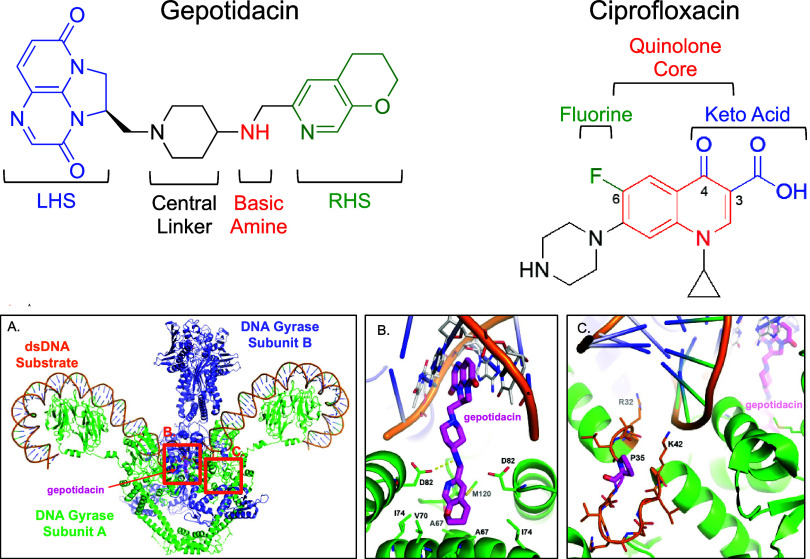
Structures of the triazaacenaphthylene gepotidacin and the fluoroquinolone ciprofloxacin and the positioning of gepotidacin in the active site of *E. coli* gyrase. The key pharmacophoric elements of gepotidacin are shown (top left): left-hand side that pi–pi stacks with two central base pairs of the stretched DNA (LHS, blue), central linker (black), basic amine that interacts with *E. coli* GyrA^D82^ (equivalent to ParC^D79^; red), and right-hand side that binds in a largely hydrophobic GyrA or ParC pocket that opens up on the dimer interface (RHS, green). The triazaacenaphthylene is the LHS moiety. The quinolone core (red), C6 fluorine (green), and C3/4 keto acid (blue) substituents of ciprofloxacin are highlighted (top right). The structure of gepotidacin bound to *E. coli* gyrase showing predicted interactions with residues GyrA^D82^ and GyrA^P35^ is shown at the bottom. (A) Structure of the ternary complex of gepotidacin, *E. coli* gyrase, and double-stranded (ds) DNA. Left and right boxes B and C show the locations of GyrA^D82^ and GyrA^P35^, respectively, relative to gepotidacin. (B) Zoomed-in image showing that gepotidacin interacts via a single hydrogen bond with GyrA^D82^. The mutation GyrA^D82N^ (ParC^D79N^) modifies the charge of the residue and directly affects the ability of the residue to hydrogen-bond with gepotidacin (pink carbon bonds). (C) Zoomed-in image showing that GyrA^P35^ is distal from gepotidacin but near the break in the DNA substrate. The GyrA^P35^ mutation possibly modifies the geometry of the loop spanning GyrA^*R*32^ and GyrA^K42^—two residues likely responsible for the proper orientation of the double-helix during DNA cleavage. Images were generated using a structure from Protein Data Bank (PDB ID 6RKW)^72^ and PYMOL software.

Recently, enrollment into gepotidacin phase III clinical trials (EAGLE-2 and EAGLE-3) for the treatment of uncomplicated urinary tract infections caused by uropathogens, including *Escherichia coli*, was stopped early for demonstrated efficacy following a recommendation by the Independent Data Monitoring Committee.^23^ These findings support the potential clinical use of gepotidacin in the treatment of human infections. In these phase III trials, “gepotidacin demonstrated noninferiority to nitrofurantoin, an existing first-line treatment for uncomplicated urinary tract infections, in patients with a confirmed uncomplicated urinary tract infection and a uropathogen susceptible to nitrofurantoin. Additionally, in the EAGLE-3 phase III trial, gepotidacin demonstrated statistically significant superiority versus nitrofurantoin.”^24^ Gepotidacin is also currently in a phase III clinical trial for the treatment of uncomplicated urogenital gonorrhea.^16^

Gepotidacin targets both DNA gyrase and topoisomerase IV. These bacterial type II topoisomerases are essential enzymes that modulate the topological state of the chromosome.^6,9,25−30^ Gyrase controls DNA under- and overwinding and works ahead of replication forks and transcription complexes to remove positive supercoils that accumulate as a result of these DNA processes. It is the only enzyme that can actively underwind (i.e., negatively supercoil) DNA.^31−34^ Although topoisomerase IV can relax negative and positive DNA supercoils, its primary function is to decatenate, or remove tangles between, daughter chromatids that are created during replication and eliminate knots that are generated during recombination.^10,28,30,35,36^ To carry out their catalytic activities, these enzymes use a double-stranded DNA passage mechanism.^6,9,28,30,36,37^ During this reaction, gyrase and topoisomerase IV generate a transient double-stranded DNA break in one segment of DNA and pass an intact double helix through the DNA gate. To maintain genomic integrity during this process, the enzymes form covalent bonds between active site tyrosine residues and terminal phosphates generated by enzyme-mediated DNA cleavage. This covalent enzyme-cleaved DNA complex is known as the cleavage complex.^6,9,28,30,36,37^

The interactions of fluoroquinolones with gyrase and topoisomerase IV stabilize cleavage complexes, leading to the accumulation of high levels of enzyme-mediated DNA breaks in the bacterial chromosome.^4,6−10^ This action induces DNA recombination and repair pathways and the SOS response. Drugs that act in this fashion are referred to as topoisomerase poisons.^6,8−10,38^ Fluoroquinolone interactions also block the overall catalytic reaction of gyrase and topoisomerase IV.^6,9,10^ This action deprives the cell of the essential catalytic activities of these two enzymes, which can impair DNA replication/transcription and leave daughter chromosomes entangled.^6,9,10^ Both effects of fluoroquinolones are capable of inducing bacterial cell death. However, at the present time, the relative contributions of DNA cleavage and enzyme inhibition to cell death induced by fluoroquinolones and other topoisomerase poisons are not well-defined.^10,39−41^

Fluoroquinolones such as ciprofloxacin (Figure 1) interact with bacterial type II topoisomerases through a water–metal ion bridge that is anchored by the C3/C4 keto acid on the fluoroquinolone skeleton and two highly conserved amino acid residues in the GyrA/ParC subunits of gyrase and topoisomerase IV, respectively.^6,9,42−45^ These residues were first described as Ser83 in the A subunit of *E. coli* gyrase and an acidic residue (Asp87) that is four amino acids away from Ser83.^46−48^ Fluoroquinolone resistance is characterized by mutations in the anchoring amino acid residues.^6,8−10^ This resistance is further facilitated by two issues. First, most mutations in the amino acids that anchor the water–metal ion bridge retain high catalytic activity^44,49,50^ and do not affect the viability of the bacteria in the absence of drugs.^47^ Second, although both gyrase and topoisomerase IV are targeted by fluoroquinolones, in the vast majority of species, this targeting is not well-balanced. In most cases, gyrase is the primary cytotoxic target.^6,9,10,39,47^ Consequently, a single point mutation in gyrase is often sufficient to induce a level of fluoroquinolone resistance that impairs clinical efficacy and allows further resistance mutations to accumulate.^6,9,10,39,47^

Like the fluoroquinolones, gepotidacin and other novel bacterial topoisomerase inhibitors (NBTIs) stabilize the cleavage complex^42,51−57^ and inhibit overall enzyme activity.^42,51−69^ However, in contrast to drugs like ciprofloxacin, gepotidacin and other NBTIs interact with different residues on gyrase and topoisomerase IV^42,54,70−72^ and induce enzyme-mediated single-stranded, as opposed to double-stranded, DNA breaks.^52−57^ Although two fluoroquinolone molecules bind in the cleavage complex, inserting at the sites of DNA cleavage on both strands of the double-helix,^42,43,45,54,73,74^ like other NBTIs, a single gepotidacin molecule binds midway between the two scissile DNA bonds in a pocket between the two A subunits of the bacterial type II enzymes.^11,42,54,70−72^

A previous study that characterized the effects of gepotidacin on *S. aureus* gyrase found that the compound shares many mechanistic features with earlier preclinical candidate novel bacterial topoisomerase inhibitors.^42,54,72^ However, virtually nothing is known regarding the actions of gepotidacin on topoisomerase IV.^42,51−69^ Because of the clinical promise of gepotidacin against urinary tract infections, it is important to characterize how this compound affects the activities of both gyrase and topoisomerase IV in *E. coli*. Moreover, the roles that the two enzymes play in the antibacterial activity of the compound and the mechanism of target-mediated resistance need to be further clarified. Consequently, we examined the antibacterial activity of gepotidacin against *E. coli* strains and the effects of gepotidacin on wild-type (WT) and mutant gyrase and topoisomerase IV. Gepotidacin displayed well-balanced dual-targeting in cells, which is consistent with a potential low propensity for target-mediated resistance in this species.^75^ The compound inhibited the DNA supercoiling and decatenation activities of purified WT gyrase and topoisomerase IV, respectively, with similar sub-μM IC_50_ values, further supporting the well-balanced targeting of these enzymes. Under a variety of conditions, gepotidacin enhanced only single-stranded DNA cleavage mediated by gyrase and topoisomerase IV. Finally, mutations in *E. coli* gyrase and topoisomerase IV amino acid residues that are involved in gepotidacin–target interactions diminished the activity of the compound against both enzymes. These results provide valuable insights into the mechanism of action of this clinically important first-in-class triazaacenaphthylene antibacterial.

## Results

### Gepotidacin Displays Balanced Dual-Targeting of Gyrase and Topoisomerase IV in *E. coli* Cells

Drugs that act against both gyrase and topoisomerase IV, such as fluoroquinolones, can differentially target these enzymes with regard to antibacterial activity.^6,7,9,10^ Depending on the bacterial strain and the drug employed, gyrase or topoisomerase IV can act as the primary lethal target of fluoroquinolones.^6,9,76−78^ Although gyrase is usually the primary lethal target of these drugs, dual-targeting, in which the action of the drug against either enzyme is sufficient for lethality, has been reported.^77,78^ Drugs that equally target gyrase and topoisomerase IV have an obvious advantage in that target-mediated resistance should be observed only when mutations in both enzymes occur simultaneously. In *E. coli*, gyrase is the primary lethal target of ciprofloxacin.^47,79^

Previous studies suggest that in contrast to fluoroquinolones, some topoisomerase-targeted compounds display well-balanced dual-targeting of gyrase and topoisomerase IV in *E. coli*.^75,80^ Whereas *E. coli* cells carrying either the GyrA^D82G^ or ParC^D79G^ mutation displayed WT susceptibility toward NBTI 5463, a compound that differs from gepotidacin in the moieties utilized for interactions with DNA (left-hand side), protein-binding pocket (right-hand side), and the key aspartic acid (basic amine), cells that carried both mutations were >100-fold less susceptible to the compound.^80^ This compound has a similar binding site to gepotidacin but was not progressed to the clinic. A comparable finding was reported for gepotidacin in cells that carried the GyrA^D82N^ and ParC^D79N^ mutations.^75^ Although the MIC (minimum inhibitory concentration) value of the compound against cells carrying the individual mutations was within twofold of that of the WT strain, the MIC value in cells carrying both mutations rose to more than 256 times that of WT.

To further explore the potential well-balanced dual-targeting of gepotidacin in *E. coli*, we examined strains that encoded the GyrA^P35L^ and ParC^D79N^ mutations. Gepotidacin is bactericidal against *E. coli* cells^81^ and was lethal to the *E. coli* strains used in the present study. The compound displayed an MIC of 0.125 μg/mL against the parental TOP10 WT strain (Table 1).

**Table 1:** Gepotidacin Displays Dual-Targeting of Gyrase and Topoisomerase IV in *E. coli* Cells^a^

	mutation		MIC (μg/mL)			
*E. coli*	GyrA	ParC	gepotidacin	fold	ciprofloxacin	fold
TOP10	WT	WT	0.125	NA	0.0015	NA
TOP10-1	P35L	WT	0.125	1	0.012	8
TOP10-2	WT	D79N	0.125	1	0.002	1
TOP10-3	P35L	D79N	16	128	0.012	8

a The minimum inhibitory concentrations (MICs) of gepotidacin are shown for four isogenic *E. coli* strains: TOP10 (GyrA^WT^, ParC^WT^), TOP10-1 (GyrA^P35L^, ParC^WT^), TOP10-2 (GyrA^WT^, ParC^D79N^), and TOP10-3 (GyrA^P35L^, ParC^D79N^). The fold change from the WT strain (TOP10) is also shown. Ciprofloxacin MICs and fold changes are shown for comparison. NA = nonapplicable.

Unlike fluoroquinolones, no spontaneous individual mutations in *E. coli* gyrase or topoisomerase IV that resulted in reduced susceptibility to gepotidacin were generated in cellular in vitro frequency of resistance studies.^82^ Therefore, to further explore the antibacterial activity of gepotidacin, we generated an *E. coli* TOP10 strain that carried the ParC^D79N^ mutation. On the basis of structural studies, ParC^D79^ is predicted to interact with the basic amine group of gepotidacin.^54,72,83^ The presence of ParC^D79N^ increased the frequency of spontaneous resistance to an earlier related compound, GSK203815, under selective pressure. This allowed for the construction of a strain carrying mutations in both gyrase and topoisomerase IV (GyrA^P35L^ and ParC^D79N^, respectively). Structural and modeling studies predict that GyrA^P35^ may be involved in DNA binding/bending.^83^ Consequently, we engineered three isogenic *E. coli* TOP10 strains, TOP10-1 (GyrA^P35L^), TOP10-2 (ParC^D79N^), and TOP10-3 (GyrA^P35L^, ParC^D79N^) (Table 1), to evaluate their susceptibility to gepotidacin.

The MIC values for gepotidacin against the strains carrying individual mutations in gyrase (TOP10-1) or topoisomerase IV (TOP10-2) were the same as that seen with the WT TOP10 parent strain (MIC = 0.125 μg/mL). However, the MIC value for gepotidacin against the TOP10-3 strain that carried mutations in both enzymes was 128-fold higher (MIC = 16 μg/mL). These data suggest that the GyrA^P35L^ and ParC^D79N^ mutations confer decreased target sensitivity to gepotidacin but that simultaneous mutations in both enzymes are required for reduced cellular susceptibility to the compound. Thus, it appears that gyrase and topoisomerase IV are dual-targets for gepotidacin in *E. coli* and that this compound targets both enzymes in a well-balanced fashion (i.e., gepotidacin is equally capable of inhibiting the growth of *E. coli* cells through its actions against either gyrase or topoisomerase IV).

*E. coli* TOP10-1 cells carrying the GyrA^P35L^ mutation displayed an MIC value for ciprofloxacin that was eightfold higher than that observed with WT cells (Table 1; MIC = 0.012 μg/mL compared to the WT MIC of 0.0015 μg/mL). Little or no change was observed in the TOP10-2 strain that carried the topoisomerase IV ParC^D79N^ mutation alone (MIC = 0.002 μg/mL), and fluoroquinolone susceptibility in the TOP10-3 double mutant strain was similar to that of the singly mutated TOP10-1 strain. These results are in line with gyrase being the primary cytotoxic target of fluoroquinolones in *E. coli*. Consistent with an earlier study,^75,80^ these findings indicate that fluoroquinolones may still maintain efficacy against some strains carrying mutations that affect cellular susceptibility to gepotidacin.

### Gepotidacin Is a Potent Catalytic Inhibitor of WT *E. coli* Gyrase and Topoisomerase IV

The cellular studies provide strong evidence that gyrase and topoisomerase IV are both targeted by gepotidacin in a well-balanced fashion. However, the effects of gepotidacin on *E. coli* type II enzymes have never been reported. Therefore, we examined the effects of gepotidacin on the catalytic activities of WT *E. coli* gyrase and topoisomerase IV.

**Figure 2:**
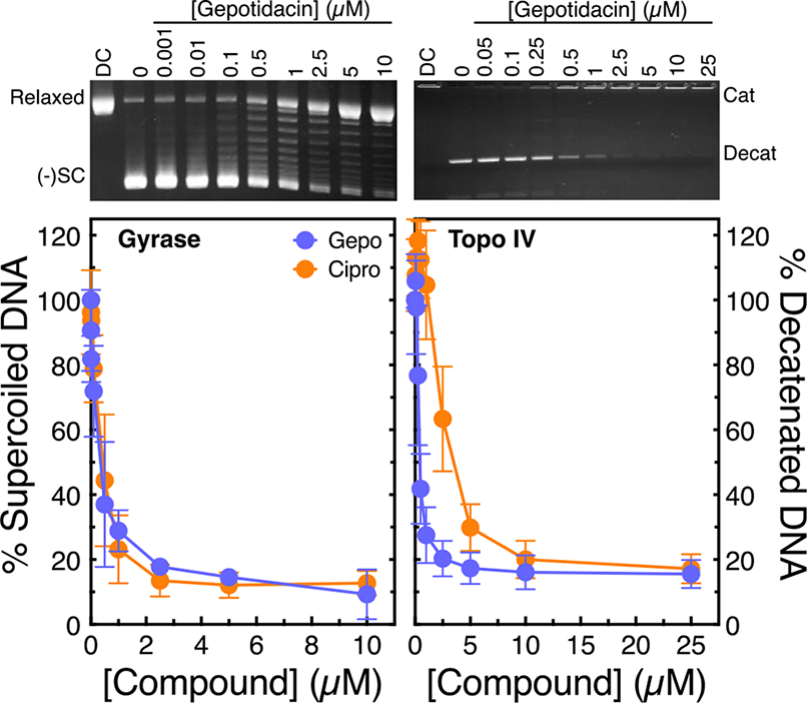
Gepotidacin is a potent inhibitor of WT *E. coli* gyrase-catalyzed DNA supercoiling and WT topoisomerase IV-catalyzed DNA decatenation. The effects of gepotidacin (blue) and ciprofloxacin (orange) on DNA supercoiling and decatenation mediated by gyrase (left) and topoisomerase IV (right), respectively, are shown. Error bars represent the standard deviation (SD) of at least three independent experiments. The gels shown at the top are representative of supercoiling (left) and decatenation (right) assays with gepotidacin. DC represents the fully relaxed (left) or fully catenated (right) DNA control. The mobilities of relaxed, negatively supercoiled, [(−)SC], catenated (Cat), and decatenated (Decat) DNA are indicated.

Gepotidacin inhibited DNA supercoiling (i.e., the conversion of relaxed to negatively supercoiled plasmid) catalyzed by *E. coli* gyrase at submicromolar concentrations (IC_50_ = 0.32 ± 0.17 μM) (Figure 2, left panel, blue) (a summary of data for gepotidacin is shown in Table 2). Gepotidacin was also a potent inhibitor of topoisomerase IV-catalyzed decatenation (IC_50_ = 0.34 ± 0.09 μM) (Figure 2, right panel, blue; Table 2). The similar IC_50_ values for the inhibition of both enzyme activities support the well-balanced dual-targeting of gepotidacin in *E. coli* cells.

**Table 2:**
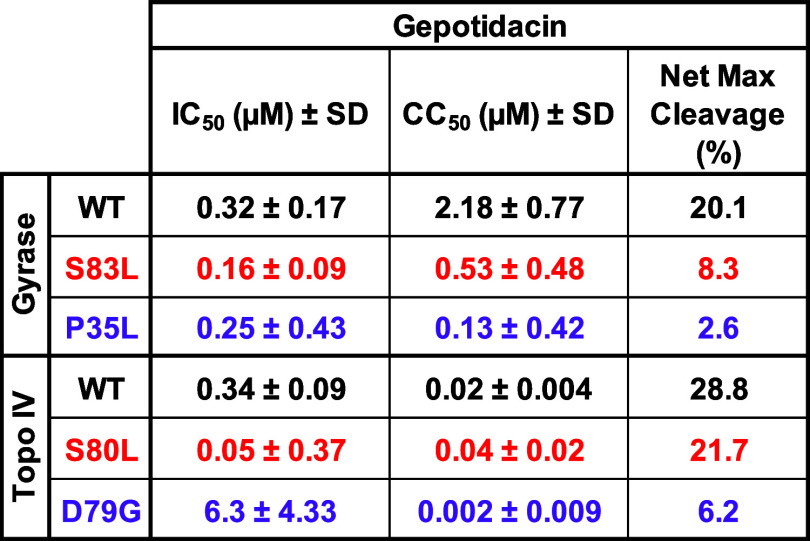
Summary of IC_50_ and CC_50_ Values for Gepotidacin against WT *E. coli* Gyrase and Topoisomerase IV, Mutant Gyrase Enzymes that Carry the GyrA^S83L^ (S83L) or GyrA^P35L^ (P35L) Mutation, or Mutant Topoisomerase IV Enzymes that Carry the ParC^S80L^ (S80L) or ParC^D79G^ (D79G) Mutation^a^

a IC_50_ values with gyrase and topoisomerase IV were calculated on the basis of DNA supercoiling and decatenation assays, respectively. Standard deviations (SD) are shown. Net Max Cleavage represents the maximal single-stranded DNA cleavage after subtracting the baseline levels of DNA cleavage.

In Figure 2, it is not obvious why gepotidacin and ciprofloxacin do not suppress 100% of enzyme catalysis. However, it is likely because the DNA supercoiling and decatenation assays are steady state rather than equilibrium (as in the DNA cleavage assays), and any gyrase or topoisomerase IV molecule that completes its catalytic cycle before a drug molecule binds or following drug dissociation will result in an apparent low level of remaining activity that cannot be overcome.

Despite the fact that ciprofloxacin displayed MIC values lower than those of gepotidacin against *E. coli* cells (Table 1), this enhanced antibacterial activity was not reflected in in vitro enzyme assays. The IC_50_ value for ciprofloxacin in gyrase-catalyzed supercoiling assays (0.33 ± 0.12 μM) (Figure 2, left panel, orange) was similar to that observed for gepotidacin. In addition, ciprofloxacin was less potent against topoisomerase IV-catalyzed decatenation (i.e., the conversion of a catenated network of DNA circles to monomers) (IC_50_ = 2.47 ± 0.48 μM) (Figure 2, right panel, orange; Table 2) consistent with gyrase being the primary target of ciprofloxacin in *E. coli*. It is not known why the MIC values for ciprofloxacin against *E. coli* cells are lower than those of gepotidacin. It could be due to enhanced uptake or decreased efflux or differences in the metabolism of the fluoroquinolone and the triazaacenaphthylene. Alternatively, fluoroquinolone-induced DNA damage may be more lethal to bacterial cells.

### Gepotidacin Induces Single-Stranded DNA Breaks Generated by WT *E. coli* Gyrase and Topoisomerase IV

**Figure 3:**
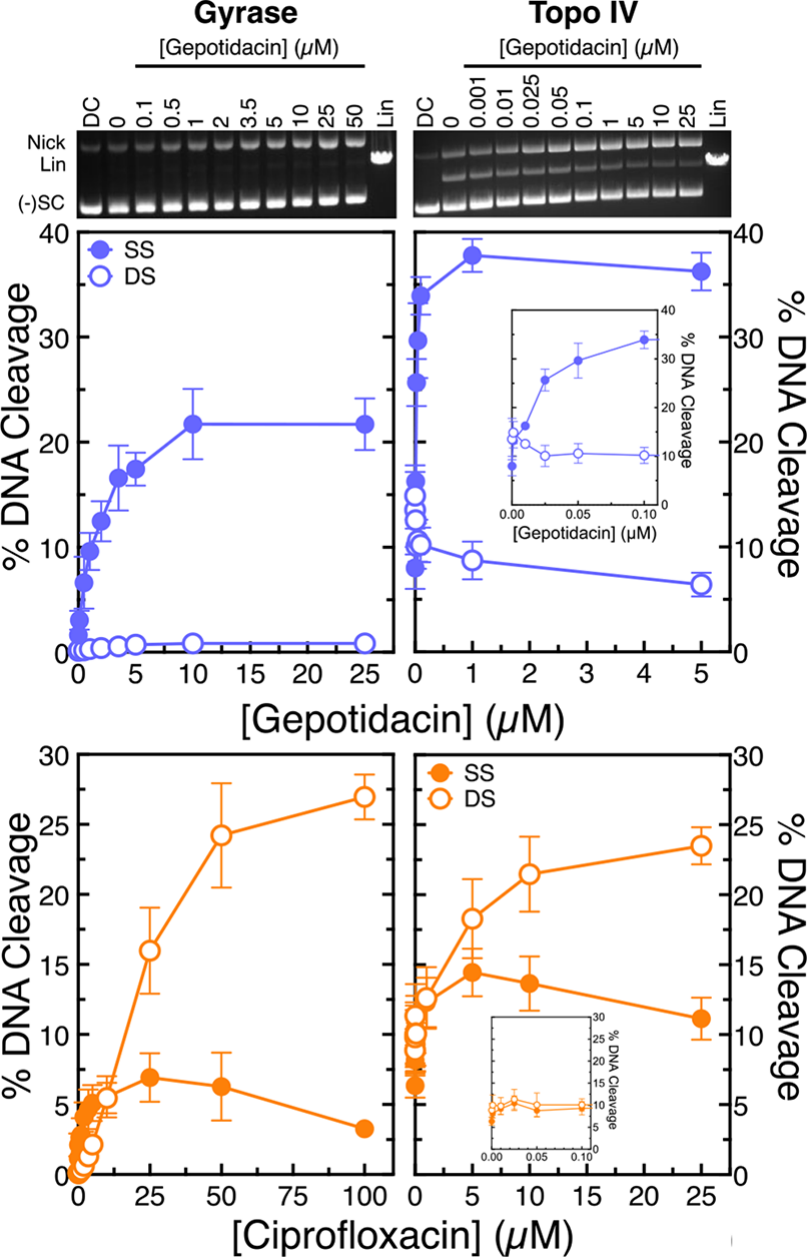
Gepotidacin enhances single-stranded DNA breaks mediated by WT *E. coli* gyrase and topoisomerase IV. The effects of gepotidacin (blue, top) and ciprofloxacin (orange, bottom) on DNA cleavage mediated by gyrase (left) and topoisomerase IV (Topo IV, right) are shown. Levels of single-stranded (SS, closed circles) and double-stranded (DS, open circles) DNA breaks are shown. Error bars represent the SD of at least three independent experiments. The insets show the effects of low concentrations of gepotidacin and ciprofloxacin on single- and double-stranded DNA cleavage mediated by WT topoisomerase IV. The gels shown at the top are representative DNA cleavage assays with gepotidacin. The mobilities of nicked (Nick or SS), linear (Lin or DS), and negatively supercoiled [(−)SC] are indicated.

Gepotidacin is a potent enhancer of DNA cleavage [i.e., the conversion of negatively supercoiled plasmid to nicked (single-stranded scission) or linear (double-stranded scission) DNA products] mediated by *E. coli* gyrase (Figure 3). Similar to the case of other NBTIs, gepotidacin induced only single-stranded DNA breaks. CC_50_ (concentration at which 50% maximal DNA cleavage was observed) was 2.18 ± 0.77 μM, with levels of single-stranded DNA breaks maxing out at ∼20.1% of the initial DNA substrate (Figure 3, top left panel; Table 2). Gepotidacin was even more active against *E. coli* topoisomerase IV, inducing nearly 40% single-stranded DNA cleavage with a CC_50_ value of 0.02 ± 0.004 μM (Figure 3, top right panel; Table 2). Note that this high level of DNA cleavage is inflated by the high baseline levels of scission mediated by *E. coli* topoisomerase IV in the absence of drugs (∼8% single-stranded and ∼15% double-stranded DNA breaks). Again, no enhancement of the double-stranded DNA cleavage was observed.

For comparison, the effects of ciprofloxacin on gyrase/topoisomerase IV-mediated DNA cleavage are shown in Figure 3 (bottom left and right panels, respectively). As reported previously,^42,52,55,84^ ciprofloxacin induced both single- and double-stranded DNA breaks, with the latter being more prevalent. In contrast, gepotidacin strongly enhanced only single-stranded DNA breaks.

Although no enhancement of topoisomerase IV-mediated double-stranded DNA cleavage was observed in the presence of gepotidacin, the high baseline DNA cleavage activity of this enzyme allowed us to further examine the effects of gepotidacin on DNA scission. As seen in Figure 3 (top right panel), gepotidacin suppressed the ability of *E. coli* topoisomerase IV to generate double-stranded DNA breaks. This suppression has been reported previously for gepotidacin with *S. aureus* gyrase^54^ and for some other NBTIs.^53,55^ The reason for this suppression is unknown. However, it is believed that gepotidacin and related molecules induce sufficient distortion in the active site of gyrase/topoisomerase IV following cleavage of one DNA strand that it prevents the enzyme from cleaving the second strand.^42,53^ Unfortunately, the low baseline levels of DNA cleavage generated by *E. coli* gyrase (Figure 3, top left panel), even in the presence of divalent metal ions such as Ca^2+^ that often enhance enzyme-mediated DNA cleavage,^49,53,85−87^ did not allow us to examine this suppression of gyrase-generated double-stranded DNA breaks.

**Figure 4:**
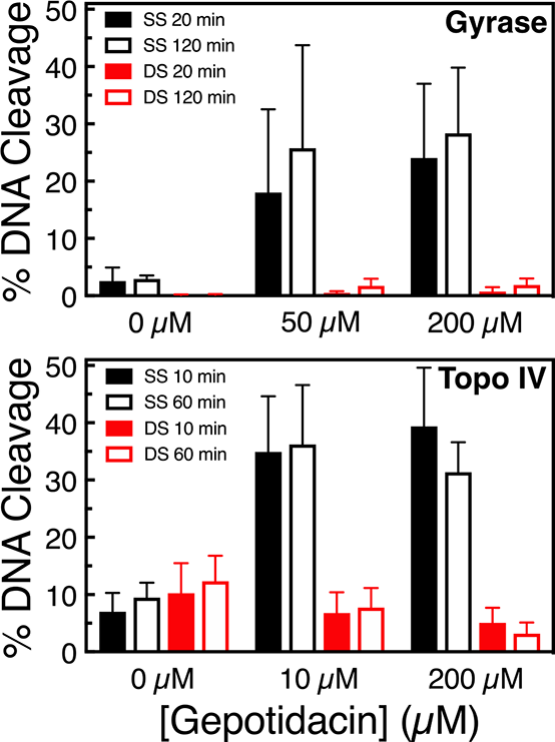
Gepotidacin enhances only single-stranded breaks mediated by WT *E. coli* gyrase and topoisomerase IV. The enhancement of gyrase-mediated single-stranded (SS, black) or double-stranded (DS, red) cleavage at 20 min (filled bar) or 120 min (open bar) in the presence of 0, 50, or 200 μM gepotidacin is shown. Bottom panel: The enhancement of topoisomerase IV-mediated single-stranded (black) or double-stranded (red) cleavage at 10 min (filled bar) or 60 min (open bar) in the presence of 0, 10, or 200 μM gepotidacin is shown. Error bars represent the SD of at least three independent experiments.

Two additional experiments were carried out to further assess the effects of gepotidacin on single-stranded gyrase/topoisomerase IV-mediated DNA cleavage. In the first, the ability of gepotidacin to induce single- versus double-stranded DNA scission at high concentrations and at long time courses was examined (Figure 4). The conditions used for gyrase (top panel) were 50 and 200 μM gepotidacin (5 and 20 times the concentration necessary for maximal cleavage) at incubation times of 20 and 120 min (one- and sixfold longer than a normal cleavage assay). The conditions used for topoisomerase IV (bottom panel) were 10 and 200 μM gepotidacin (10 and 200 times the concentration necessary for maximal cleavage) at incubation times of 10 and 60 min (one- and sixfold longer than a normal cleavage assay). Under all circumstances, only single-stranded DNA breaks were induced by gepotidacin.

**Figure 5:**
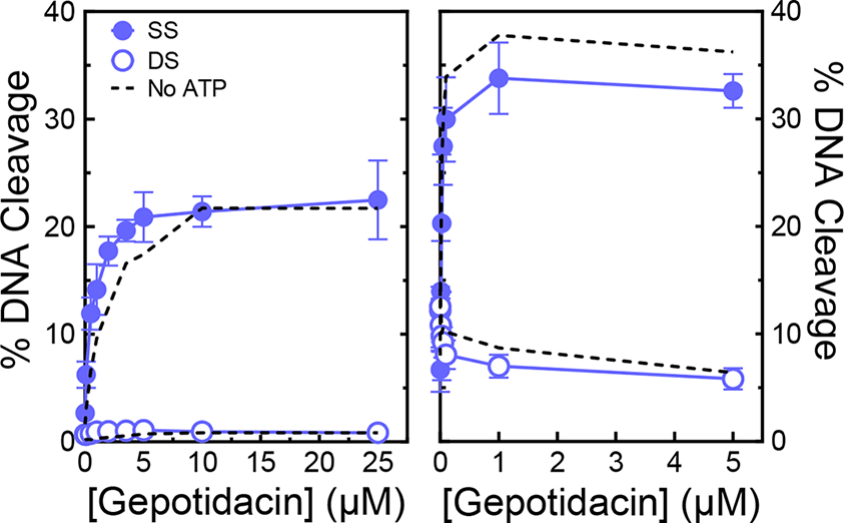
Gepotidacin enhances only single-stranded breaks mediated by WT *E. coli* gyrase and topoisomerase IV in the presence of ATP. The effects of ATP on gepotidacin-induced single- (SS, closed blue circles) and double-stranded (DS, open blue circles) breaks mediated by gyrase (left panel) and topoisomerase IV (right panel) are shown. DNA cleavage in the absence of ATP (Figure 3) is shown as dashed lines for comparison. Error bars represent the SD of at least three independent experiments.

In the second experiment, the effects of 1.5 mM ATP on DNA cleavage were examined. Although the high energy cofactor is not required for DNA cleavage, it is necessary to support the overall catalytic activity of gyrase and topoisomerase IV.^9,28,88,89^ ATP binding drives the closing of the N-terminal protein gate and the DNA strand passage event, whereas ATP hydrolysis drives enzyme turnover.^9,28,88,89^ In some cases, ATP has enhanced the effects of topoisomerase poisons on the stimulation of DNA scission, and in others, it has impaired the actions of these compounds.^41,55^ As seen in Figure 5, ATP had little effect on the ability of gepotidacin to induce DNA cleavage by *E. coli* gyrase (left panel) or topoisomerase IV (right panel). Levels of single-stranded cleavage were maintained; no double-stranded break enhancement was observed; and suppression of double-stranded DNA cleavage by *E. coli* topoisomerase IV was seen. Therefore, in the bacterial cell, which contains millimolar concentrations of ATP,^90^ gepotidacin should maintain its activity against gyrase and topoisomerase IV-mediated DNA cleavage and induce only enzyme-mediated single-stranded DNA breaks.

### Gepotidacin Induces Stable DNA Cleavage Complexes with WT *E. coli* Gyrase and Topoisomerase IV

**Figure 6:**
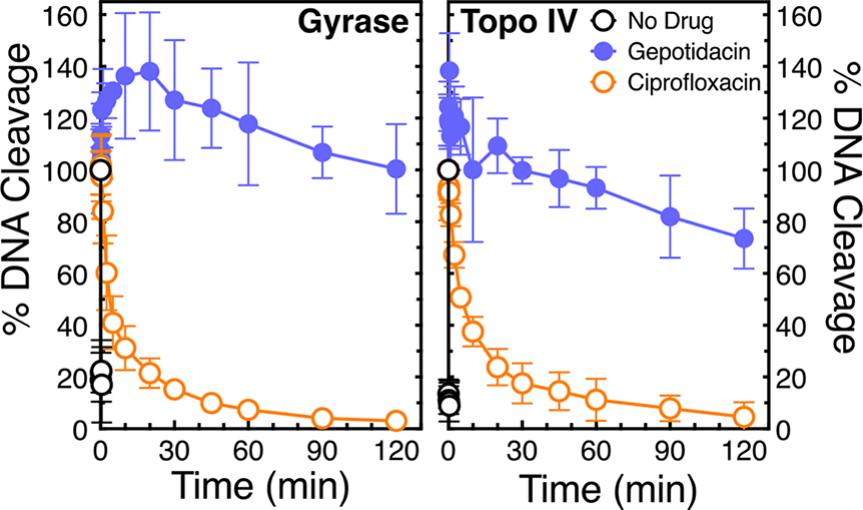
Gepotidacin induces stable single-stranded DNA breaks generated by WT *E. coli* gyrase and topoisomerase IV. The persistence of DNA cleavage complexes mediated by gyrase (left) is shown in the absence of the compound (black, open circle) or in the initial presence of 10 μM gepotidacin (blue, closed circle) or 50 μM ciprofloxacin (orange, open circle). Gyrase and plasmid concentrations in initial assay mixtures were 500 and 50 nM, respectively. The persistence of DNA cleavage complexes mediated by topoisomerase IV (right panel) is shown in the absence of compound (black, open circle) or in the initial presence of 0.1 μM gepotidacin (blue, closed circle) or 10 μM ciprofloxacin (orange, open circle). Topoisomerase IV and plasmid concentrations in initial assay mixtures were 100 and 50 nM, respectively. Initial reaction mixtures contained 5 mM MgCl_2_. Assays were initiated by the 20-fold dilution of reaction mixtures into buffer that lacked divalent cation. Double-stranded DNA breaks generated in the absence of a compound or in the presence of ciprofloxacin are shown as open circles, while single-stranded breaks generated in the presence of gepotidacin are shown as closed circles. DNA cleavage at time 0 was set to 100%. Error bars represent the SD of at least three independent experiments.

In general, drugs that induce more stable cleavage complexes are more lethal to cells.^91^ Therefore, we used a persistence assay^44,91,92^ to assess the stability of gyrase– and topoisomerase IV–DNA cleavage complexes formed in the presence of gepotidacin. In this assay, ternary enzyme–DNA–drug complexes were formed at high concentrations of enzyme and DNA (and when present, saturating concentrations of gepotidacin or ciprofloxacin; see Figure 6 legend for details) and then diluted 20-fold into buffer that did not contain the divalent cation necessary to sustain DNA cleavage. Following dilution, ternary complexes that dissociate are highly unlikely to reform.^44,91,92^ The stability of these complexes is monitored by the loss of single-stranded DNA cleavage generated in the presence of gepotidacin and compared with double-stranded DNA cleavage in the absence of compounds or in the presence of ciprofloxacin. As seen in Figure 6, gepotidacin induced the formation of very stable cleavage complexes with *E. coli* gyrase (left panel) and topoisomerase IV (right panel). With both enzymes, the half-life (*T*_1/2_) was well in excess of 120 min. In contrast, in the absence of the drug, the *T*_1/2_ of cleavage complexes was less than 5 s. Postdilution times for the data points for cleavage complexes formed in the absence of drug in Figure 6 ranged between 5 and 15 s. Levels of cleavage complex remaining at 5 s in the absence of drug were 18.9 and 13.6% for gyrase and topoisomerase IV, respectively. A previous rapid quench kinetic study estimated that the half-life of cleavage complexes formed with *E. coli* gyrase and topoisomerase IV in the absence of drugs was 0.16 and 0.21 s, respectively.^93^ Ciprofloxacin stabilized cleavage complexes compared to the no-drug experiments (*T*_1/2_ = 3.6 and 5.7 min with gyrase and topoisomerase IV, respectively). However, the lifetimes of these complexes were substantially shorter than those seen with gepotidacin.

### Gepotidacin Competes with Ciprofloxacin for Activity Against WT *E. coli* Gyrase and Topoisomerase IV

Crystallography and cryo-EM structural studies place gepotidacin and fluoroquinolones in the active site of gyrase and topoisomerase IV.^9,42,43,45,72,94^ Two fluoroquinolone molecules interact in the cleavage complex and insert at sites of cleavage on both strands of the double-helix. In contrast, a single gepotidacin molecule binds midway between the two scissile DNA bonds in a pocket between the two A subunits of the bacterial type II enzymes. Although fluoroquinolones and gepotidacin do not interact with the same amino acid residues, modeling and competition studies suggest that these two antibacterial classes cannot occupy the gyrase/topoisomerase IV active site at the same time.^54^ Similar results have been reported for fluoroquinolones and other NBTIs.^53,54^

**Figure 7:**
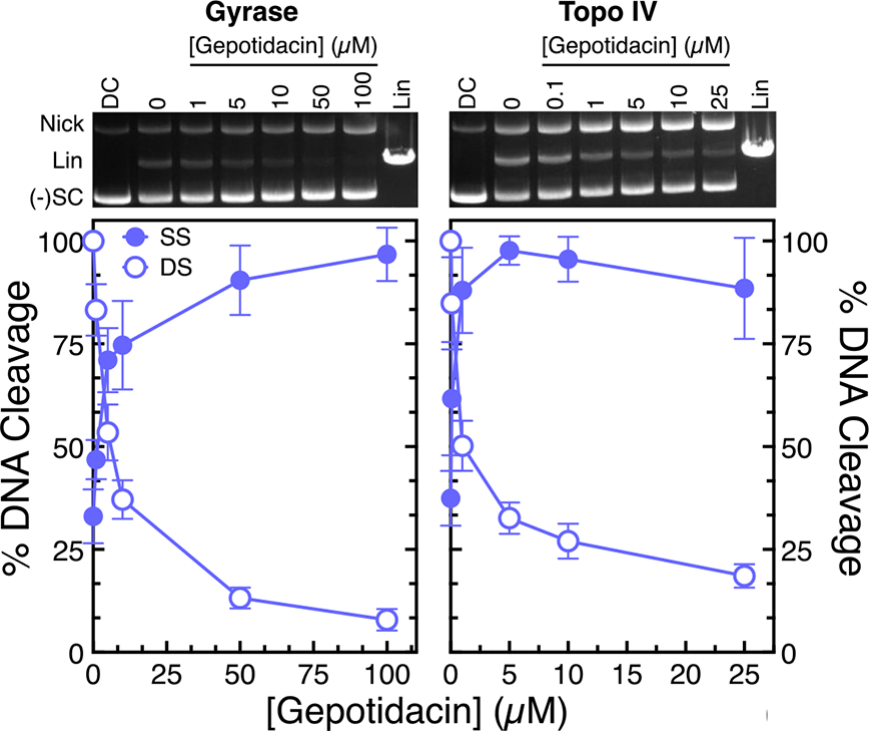
Gepotidacin competes against ciprofloxacin for the DNA cleavage/ligation active site of WT *E. coli* gyrase and topoisomerase IV. Assays with gyrase (left) contained 50 μM ciprofloxacin and 0–100 μM gepotidacin. Assays with topoisomerase IV (right panel) contained 10 μM ciprofloxacin and 0–25 μM gepotidacin. The disappearance of ciprofloxacin-induced double-stranded breaks (DS, open circles) and the appearance of gepotidacin-induced single-stranded breaks (SS, closed circles) are shown. Levels of ciprofloxacin-induced double-stranded breaks in the absence of gepotidacin were set to 100%, as were maximal levels of gepotidacin-induced single-stranded breaks. Error bars represent the SD of at least three independent experiments. The gels shown at the top are representative DNA cleavage competition assays with gepotidacin. The mobilities of nicked (Nick), linear (Lin), and negatively supercoiled [(−)SC] are indicated.

Therefore, to determine whether gepotidacin and fluoroquinolones can simultaneously act on *E. coli* gyrase and topoisomerase IV, competition studies were carried out between gepotidacin and ciprofloxacin (Figure 7). In this assay, cleavage complexes with gyrase or topoisomerase IV were formed in the presence of a mixture of 50 or 10 μM ciprofloxacin, respectively, and increasing concentrations of gepotidacin (0–100 or 0–25 μM, respectively). The levels of fluoroquinolone used in these assays reflect the concentrations at which ciprofloxacin induced maximal levels of double-stranded breaks. Because gepotidacin induces only single-stranded breaks, competition was monitored by the loss of double-stranded DNA breaks, which could have been induced only by ciprofloxacin. The increase in gepotidacin-induced single-stranded DNA cleavage also was monitored.

Gepotidacin was a potent competitor of ciprofloxacin with both gyrase (Figure 7, left panel) and topoisomerase IV (right panel) and decreased levels of double-stranded DNA breaks by 50% at concentrations of 5.66 ± 2.7 and 0.66 ± 0.97 μM, respectively. These values are ∼3- and ∼33-fold higher than the CC_50_ values for gepotidacin against the two enzymes (2.18 ± 0.77 μM for gyrase and 0.02 ± 0.004 μM for topoisomerase IV; Table 2), respectively, suggesting that the decrease in double-stranded breaks results from the competition between gepotidacin and ciprofloxacin rather than a suppression of double-stranded breaks by gepotidacin.

The decrease in ciprofloxacin-induced double-stranded breaks was accompanied by a concomitant rise in single-stranded breaks induced by gepotidacin (CC_50_ values for gepotidacin-induced DNA cleavage in the presence of ciprofloxacin were 4.63 ± 3.7 and 0.13 ± 0.09 μM for gyrase and topoisomerase IV, respectively). These values are ∼2- and ∼6-fold higher than the respective CC_50_ values for gepotidacin calculated in the absence of ciprofloxacin (Table 2). Taken together, these results indicate that gepotidacin and ciprofloxacin cannot simultaneously act on the *E. coli* type II topoisomerases and confirm that the affinity of gepotidacin for these enzymes is considerably higher than that of the fluoroquinolone.

### Effects of the *E. coli* GyrA^P35L^ Enzyme Mutation on Gepotidacin Activity

Virtually nothing is known about the biochemical mechanisms by which cells lose their susceptibility to gepotidacin. A previous report indicated that NBTI 5463 was much less potent against the ATPase activities of gyrase or topoisomerase IV that carried the GyrA^D82G^ or ParC^D79G^ mutation, respectively. However, the basis for this decreased potency was never examined. To address the important issue of resistance, we characterized the interactions of gepotidacin with two enzymes that carried mutations in amino acids that are involved in gepotidacin–target interactions (Figure 1). Gyrase GyrA^P35L^ was designed to include the mutation that was encoded in the TOP10-1 *E. coli* strain discussed earlier. Formation of inclusion bodies under conditions of overexpression prevented purification of the mutant topoisomerase IV gyrase (ParC^D79N^) that was encoded by the TOP10-2 *E. coli* strain. Therefore, the topoisomerase IV ParC^D79G^ mutant enzyme was engineered for comparative studies.

**Figure 8:**
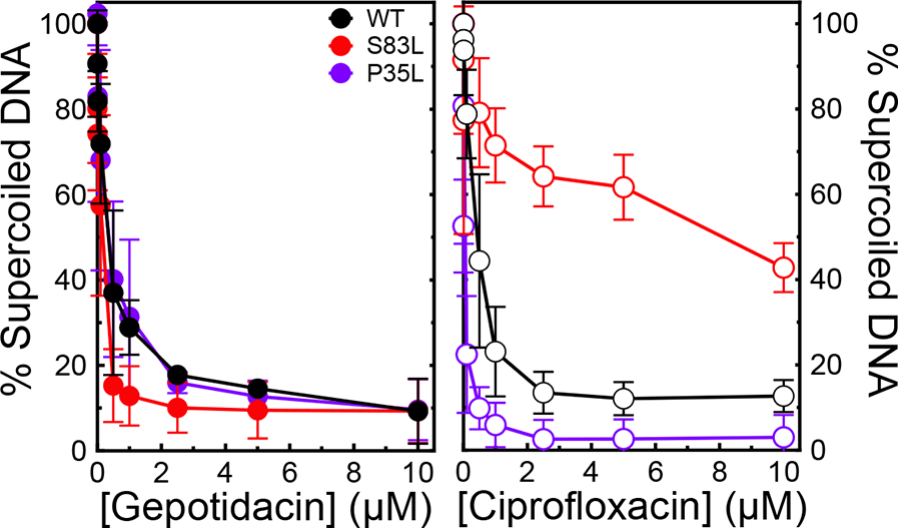
Effects of gepotidacin and ciprofloxacin on DNA supercoiling catalyzed by WT *E. coli* gyrase, the fluoroquinolone-resistant mutant GyrA^S83L^ enzyme, and the mutant GyrA^P35L^ enzyme. The effects of gepotidacin (left panel, closed circles) and ciprofloxacin (right panel, open circles) on DNA supercoiling mediated by WT (black), fluoroquinolone-resistant GyrA^S83L^ (red), and mutant GyrA^P35L^ (purple) gyrases are shown. Error bars represent the SD of at least three independent experiments.

The effects of gepotidacin on DNA supercoiling mediated by *E. coli* GyrA^P35L^ are shown in Figure 8 (left panel). As discussed above, GyrA^P35^ is predicted to be involved in DNA binding/bending.^83^ Even though GyrA^P35L^ is associated with decreased sensitivity in *E. coli* cells in the presence of a simultaneous ParC^D79N^ mutation, the mutation had no effect on the ability of gepotidacin to inhibit gyrase-catalyzed DNA supercoiling (IC_50_ = 0.25 ± 0.43 μM, as compared to 0.32 ± 0.17 μM for WT; Figure 8 and Table 2).

**Figure 9:**
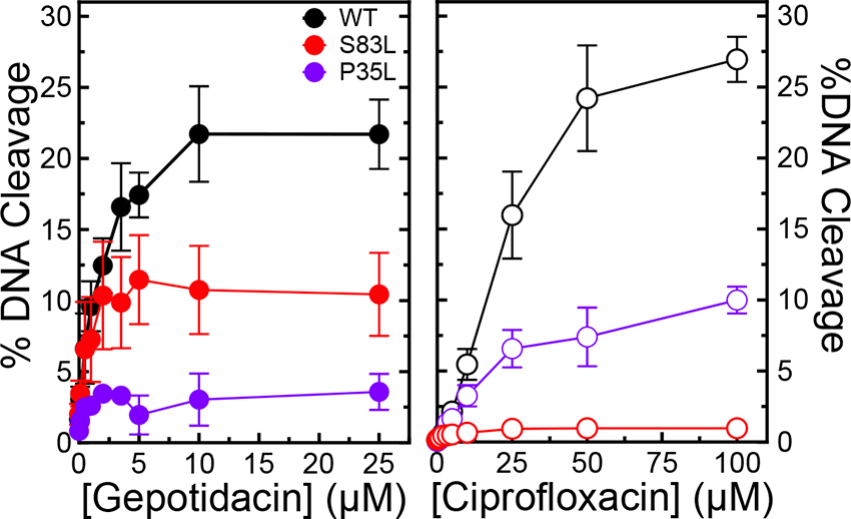
Effects of gepotidacin and ciprofloxacin on DNA cleavage mediated by WT *E. coli* gyrase, the fluoroquinolone-resistant mutant GyrA^S83L^ enzyme and the mutant GyrA^P35L^ enzyme. The enhancement of single-stranded DNA breaks in the presence of gepotidacin (left panel, closed circles) and double-stranded breaks in the presence of ciprofloxacin (right panel, open circles) mediated by WT gyrase (black), fluoroquinolone-resistant GyrA^S83L^ (red), and mutant GyrA^P35L^ (purple) are shown. Error bars represent the SD of at least three independent experiments.

In contrast, the GyrA^P35L^ mutation greatly reduced the levels of gyrase-mediated single-stranded DNA cleavage induced by gepotidacin (Figure 9, left panel; Table 2). The compound induced a maximum of ∼3.4% cleaved DNA (which is only ∼2.6% above the baseline levels of cleavage induced by the enzyme in the absence of drug) as compared to 21.7% with the WT enzyme. These findings strongly suggest that in relation to DNA gyrase, decreased cellular susceptibility to gepotidacin correlates with a diminished effect of the compound on enzyme-mediated DNA scission as opposed to its effect on the overall catalytic activity. This result further implies that the component of gepotidacin-induced cell killing that is mediated by gyrase in *E. coli* is due, at least in part, to increased levels of DNA cleavage.

Despite the fact that gepotidacin induced very little DNA cleavage with GyrA^P35L^, the CC_50_ value with the mutant enzyme (0.13 ± 0.42 μM) was even lower than that observed with WT *E. coli* gyrase (2.18 ± 0.77 μM) (Table 2). Thus, the reduction in the bactericidal activity of gepotidacin associated with the GyrA^P35L^ mutation results from a lower efficacy (i.e., maximal level of DNA cleavage) of the compound rather than a loss of potency (i.e., decrease in affinity for gepotidacin). This finding implies that the GyrA^P35L^ mutation does not diminish the level of binding of gepotidacin to the gyrase–DNA complex. Coupled with the fact that gepotidacin maintains its ability to inhibit DNA supercoiling by the GyrA^P35L^ enzyme, this mutation likely alters the positioning of gepotidacin in the twofold axis of the enzyme.

As reported previously,^42,95−99^ mutations in GyrA^S83^ greatly impair the ability of ciprofloxacin to inhibit DNA supercoiling (IC_50_ = 8.1 ± 1.67 μM as compared to WT 0.33 ± 0.12 μM) (Figure 8, right panel) and abrogate fluoroquinolone-induced DNA cleavage (Figure 9, right panel). In contrast to the fluoroquinolone, the GyrA^S83L^ mutation had little effect on the supercoiling inhibition by gepotidacin (Figure 8, left panel). In fact, the IC_50_ (0.16 ± 0.09 μM) with the mutant enzyme was slightly lower than that with WT gyrase (IC_50_ = 0.32 ± 0.17 μM) (Table 2). The GyrA^S83L^ mutation decreased gepotidacin-induced single-stranded DNA cleavage by more than 50% (maximal cleavage, 8.3 vs 20.1% for WT) (Figure 9, left panel, Table 2). Taken together, these findings predict that gepotidacin should maintain at least some activity against strains that encode the GyrA^S83L^ fluoroquinolone-resistant mutant.

Finally, ciprofloxacin maintained a high potency for inhibition of supercoiling with the GyrA^P35L^ mutant enzyme (IC_50_ = 0.08 ± 0.32 μM, Figure 8, right panel) but displayed a reduced ability to induce DNA cleavage (Figure 9, right panel). This likely accounts for the eightfold increase in MIC values for ciprofloxacin in the *E. coli* strain (TOP10-1) harboring the GyrA^P35L^ mutation compared to the wild-type strain (Table 1).

### Effects of the *E. coli* ParC^D79G^ Enzyme Mutation on Gepotidacin Activity

**Figure 10:**
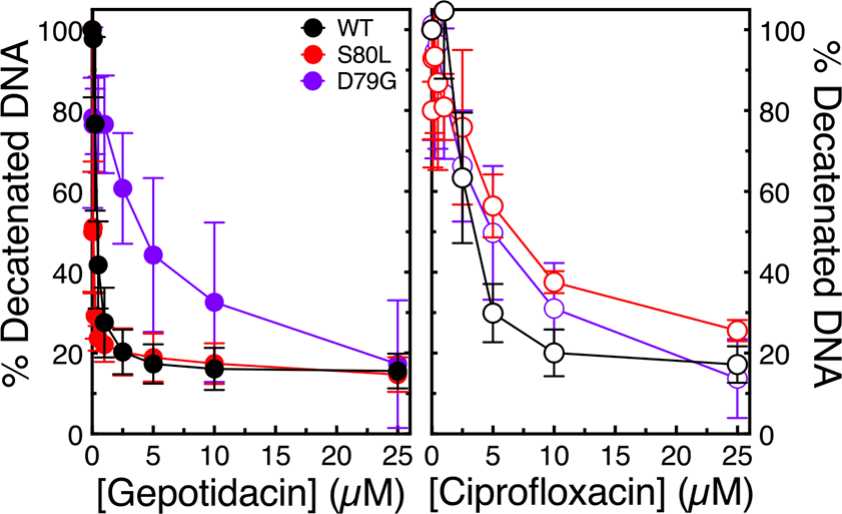
Effects of gepotidacin and ciprofloxacin on DNA decatenation catalyzed by WT topoisomerase IV, the fluoroquinolone-resistant ParC^S80L^ enzyme, and the mutant ParC^D79G^ enzyme. The effects of gepotidacin (left panel, closed circles) and ciprofloxacin (right panel, open circles) on DNA decatenation mediated by WT topoisomerase IV (black), fluoroquinolone-resistant ParC^S80L^ (red), and ParC^D79G^ (purple) are shown. Error bars represent the SD of at least three independent experiments.

**Figure 11:**
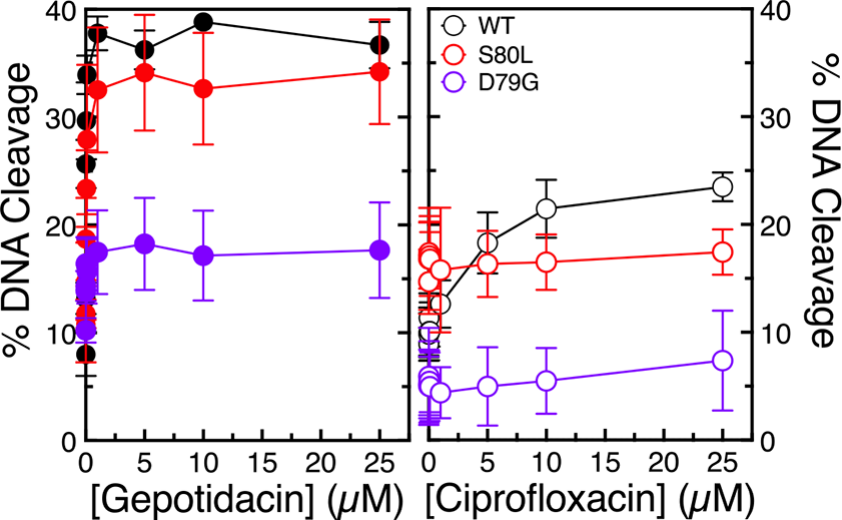
Effects of gepotidacin and ciprofloxacin on DNA cleavage mediated by *E. coli* topoisomerase IV, the fluoroquinolone-resistant mutant ParC^S80L^ enzyme, and the mutant ParC^D79G^ enzyme. The enhancement of single-stranded DNA breaks in the presence of gepotidacin (left panel, closed circles) and double-stranded breaks in the presence of ciprofloxacin (right panel, open circles) mediated by WT topoisomerase IV (black), fluoroquinolone-resistant ParC^S80L^ (red), and ParC^D79G^ (purple) is shown. Error bars represent the SD of at least three independent experiments.

The effects of gepotidacin on DNA decatenation mediated by *E. coli* ParC^D79G^ are shown in Figure 10 (left panel) and Table 2. Topoisomerase IV ParC^D79^ is predicted to interact directly with gepotidacin.^54,72,83^ The presence of the ParC^D79G^ mutation reduced the sensitivity to gepotidacin in this assay by ∼15-fold (IC_50_ = 6.30 ± 4.33 μM, as compared to 0.34 ± 0.09 μM for the WT). In addition, the mutation decreased the ability of gepotidacin to enhance topoisomerase IV-mediated single-stranded cleavage. The maximum level of single-stranded cleavage fell from ∼38% with the WT enzyme (∼28.8% above the baseline) to ∼16% (∼6.2% above the baseline) with the ParC^D79G^ mutant enzyme (Figure 11, left panel). Because both activities of gepotidacin were affected by the ParC^D79G^ mutation, it is not possible to speculate on whether the reduction in the cell-killing activity that is due to gepotidacin effects on topoisomerase IV is caused primarily by the diminished effects on DNA cleavage or the overall catalytic activity of this enzyme.

As reported previously,^100^ the ParC^S80L^ fluoroquinolone-resistant mutation decreased the ability of ciprofloxacin to inhibit DNA decatenation by ∼2.5-fold (IC_50_ = 6.3 ± 1.59 μM as compared to WT IC_50_ = 2.5 ± 0.48 μM) (Figure 10, right panel) and diminished fluoroquinolone-induced DNA cleavage by ∼4-fold (∼13% above WT baseline and ∼3% above ParC^S80L^ baseline, Figure 11, right panel). In contrast, gepotidacin was a more potent inhibitor of decatenation with the ParC^S80L^ mutant (IC_50_ = 0.05 ± 0.37 μM) (Figure 10, left panel) than against the WT enzyme (IC_50_ = 0.34 ± 0.09 μM) and maintained nearly WT levels of DNA cleavage enhancement (maximal cleavage ∼21.7% above baseline) (Figure 11, left panel). This finding suggests that cells that express the ParC^S80L^ mutation should maintain their sensitivity to gepotidacin.

Finally, ciprofloxacin partially maintained its ability to inhibit decatenation with the ParC^D79G^ mutant enzyme (IC_50_ = 5.0 ± 0.92 μM) (Figure 10, right panel) but induced considerably less DNA cleavage than that induced by the WT enzyme (maximal ParC^D79G^ cleavage of ∼2% above the baseline of ∼5%). This implies that ciprofloxacin might have a reduced activity against strains that carry this mutation. However, any decreased sensitivity would likely be obscured by the fact that topoisomerase IV is a secondary cytotoxic target for fluoroquinolones in *E. coli*.

## Discussion

Gepotidacin is a first-in-class triazaacenaphthylene antibacterial that is the most clinically advanced novel bacterial topoisomerase inhibitor.^23,113^ The compound is a potent inhibitor of *E. coli* gyrase and topoisomerase IV catalytic activity. The finding that gepotidacin displays a similar sub-μM IC_50_ value against both enzymes is consistent with its well-balanced dual-targeting in cells.

In contrast to the fluoroquinolones, gepotidacin induces only single-stranded enzyme-mediated DNA cleavage (Figures 3–5).^54,72,83^ The relative lethality of topoisomerase-induced single-stranded versus double-stranded DNA breaks has yet to be explored. However, it is well established that the stabilization of single-stranded DNA breaks by type I and II topoisomerases is sufficient to induce cell death.^101−105^

Although interactions between other novel bacterial topoisomerase inhibitors and WT gyrase and topoisomerase IV have been reported,^51−69^ the mechanisms underlying resistance to these compounds and to gepotidacin have yet to be explored. The studies presented here show that simultaneous mutations in both GyrA^P35^ and ParC^D79^ are required for a decreased susceptibility to gepotidacin in *E. coli* cells. However, individually, these mutations diminish the activity of the compound against purified gyrase and topoisomerase IV.

Gyrase/topoisomerase IV-targeted antibacterials can kill cells either by enhancing enzyme-mediated DNA cleavage or by depriving cells of essential catalytic activities.^10,39−41^ Our in vitro enzymology studies provide insights into which of these mechanisms are used by gepotidacin to kill *E. coli* cells. Indeed, the IC_50_ value of gepotidacin for supercoiling with the GyrA^P35L^ mutant was similar to that with WT gyrase, while the levels of gepotidacin-induced DNA scission were considerably lower. The finding that reduced susceptibility tracks with the diminished enhancement of DNA cleavage rather than decreased inhibition of catalytic activity implies that the mechanism by which gepotidacin induces gyrase-mediated cell death is primarily through the generation of single-stranded DNA strand breaks. In contrast to gyrase, the ParC^D79G^ mutation substantially impaired the effects of gepotidacin on both the catalytic and DNA cleavage activities of topoisomerase IV. This result makes it challenging to delineate between these two mechanisms for topoisomerase IV-mediated cell death by gepotidacin at the present time.

Finally, one of the most important attributes of gepotidacin against *E. coli* cells is its well-balanced dual-targeting. Because fluoroquinolones primarily target gyrase in *E. coli*,^47,79^ a single mutation in GyrA can result in significant resistance to fluoroquinolones. In contrast, gepotidacin requires simultaneous mutations in both gyrase and topoisomerase IV to significantly reduce the susceptibility to the compound. This well-balanced dual-targeting of gepotidacin should significantly reduce the occurrence of resistant mutant *E. coli* strains and substantially lengthen the clinical usefulness of this compound against urinary tract and other infections caused by this bacterium.

## Materials and Methods

### Construction of the Isogenic *E*. *coli* Strains with Mutations in Amino Acids that Are Involved in Gepotidacin Interactions with Gyrase and Topoisomerase IV

#### Construction of a Kanamycin-Resistant *E*. *coli* TOP10-2 (ParC^D79N^) Mutant Strain

A kanamycin-resistance marker closely linked to the *parC* gene was used to introduce the ParC^D79N^ mutation into a temperature-sensitive (ts) *E. coli* strain at a nonpermissive temperature following selection. Initially, we used *E. coli* TOP10/pKD46 competent cells grown under 0.04% L(+)-arabinose to induce the expression of λ-Red recombinase from pKD46. The cells also carried a *parC* D214G temperature-sensitive mutation (unpublished data). PCR products of approximately 5.5 kilobases (kb) were used to transform the *E. coli* TOP10/pKD46 competent cells by electroporation.^106^ These PCR products contained the gene that encoded *E. coli* ParC^D79N^ (a GAT → AAT mutation had been introduced by site-directed mutagenesis), its flanking sequences, as well as a kanamycin-resistant (KmR) cassette inserted immediately downstream of the *parC* open reading frame (ORF). The resultant transformants were selected with 50 μg/mL kanamycin on LB plates under a nonpermissive temperature of 42 °C. When the WT *parC* D214 from the PCR product was exchanged with the chromosomal *parC* D214G ts mutation by crossover, both the *parC* D79N mutation and the flanking KmR cassette were also exchanged due to close linkage to the *parC* D214. Four clones resistant to kanamycin and able to grow at 42 °C were isolated. Genetic characterization by PCR amplification and sequencing of the *parC* region from the chromosome confirmed that three clones carried the desired *parC* D79N and the KmR-cassette.

#### Isolation of a Kanamycin-Resistant *E*. *coli* TOP10-3 (GyrA^P35L^, ParC^D79N^) Double-Mutant Strain

An overnight culture of the WT *E. coli* TOP10 strain or the *E. coli* TOP10-2 (ParC^D79N^) mutant strain was plated onto LB plates containing 4X MIC of GSK203815 (an early NBTI, unpublished)^42^ and incubated at 37 °C. After incubation for 24–48 h, GSK203815-resistant colonies were isolated from the *E. coli* TOP10-2 mutant strain, but none were found in the WT *E. coli* TOP10 strain. The resultant GSK203815-resistant colonies were replated on the 4X MIC plates for further purification. Selected mutants were further characterized by MIC analysis against GSK203815 and by PCR amplification and sequencing of the GyrA and GyrB quinolone resistance-determining regions. One of the selected mutants contained the GyrA^P35L^ and ParC^D79N^ mutations.

#### Isolation of a Kanamycin-Resistant *E. coli* TOP10-1 (GyrA^P35L^) Mutant Strain

An overnight culture of WT *E. coli* TOP10 was plated onto LB plates containing 20 or 30 μg/mL of a DNA gyrase inhibitor, isoquinoline sulfonamide (IQS, cat.#: B1427, Sigma-Aldrich), and incubated at 37 °C. The *E. coli* TOP10-3 double-mutant strain described above conferred resistance to the IQS, but the *E. coli* TOP10-2 (ParC^D79N^) mutant strain did not (unpublished data). After incubation for 24 h, resistant colonies were isolated, purified, and characterized by MIC analysis against the IQS and by PCR amplification and sequencing of the GyrA quinolone resistance-determining regions. One of the IQS-resistant mutants contained the GyrA^P35L^ mutation.

#### DNA, Compounds, and Enzymes

Negatively supercoiled pBR322 DNA was prepared from *E. coli* using a Plasmid Mega Kit (Qiagen) as described by the manufacturer. Calf thymus topoisomerase I (Invitrogen) treatment of negatively supercoiled pBR322 was used to prepare relaxed plasmid DNA as described previously.^44^ Kinetoplast DNA (kDNA) was isolated from *Crithidia fasciculata* as described by Englund.^107^

Gepotidacin mesylate (GSK2140944, GlaxoSmithKline Lot no. 152392771) was stored at 4 °C as 20 mM aliquots in 100% dimethyl sulfoxide (DMSO). Ciprofloxacin was obtained from Sigma-Aldrich (cat. no. 17850, Lot no. 1396107) and stored at 4 °C as 40 mM aliquots in 0.1 N NaOH. Initial dilution (1:5) of ciprofloxacin was in 10 mM Tris-HCl, pH 7.9. ATP was obtained from Sigma-Aldrich and stored at −20 °C as 20 mM aliquots in H_2_O. All other chemicals were analytical reagent grade.

All proteins were His-tagged. WT *E. coli* gyrase subunits (GyrA and GyrB) were expressed and purified as described by Chan et al.^74^ or purchased from Profoldin (catalog ID: GDSA100 KE, Lot 112A150130). Mutant *E. coli* GyrA^S83L^ was made following site-directed mutagenesis of the WT GyrA clone and expressed and purified as described by Dong et al.^108^ WT *E. coli* topoisomerase IV subunits (ParC and ParE) and the ParC^S80L^ mutant were expressed and purified as described by Peng and Marians or by a minor modification by Corbett et al.^109,110^ Mutant *E. coli* GyrA^P35L^ and ParC^D79G^ were custom-produced by GenScript and expressed and purified as described by Chan et al.^74^

#### Determination of MIC Values

Antibacterial MIC assays were determined according to Clinical and Laboratory Standards Institute guidelines.^111^

#### Gyrase-Catalyzed DNA Supercoiling

DNA supercoiling assays were based on the procedure of Aldred et al.^112^ Assays contained 5 nM *E. coli* WT gyrase (1:1 GyrA:GyrB ratio), 5 nM relaxed pBR322, and 1.5 mM ATP in a total volume of 20 μL of 50 mM Tris-HCl (pH 7.5), 5 mM MgCl_2_, 175 mM KGlu, and 50 μg/mL bovine serum albumin (BSA). Assays with mutant enzymes contained 10 nM GyrA^S83L^ or 15 nM GyrA^P35L^. The stated enzyme concentrations reflect those of the holoenzyme (A_2_B_2_). Reactions were carried out in the absence of compound or in the presence of 0–10 μM gepotidacin or ciprofloxacin, and mixtures were incubated at 37 °C for 45 min (WT), 60 min (GyrA^S83L^), or 45 min (GyrA^P35L^), which represents the minimum time required to supercoil 80–90% of the DNA in the absence of drug. Reactions were stopped by the addition of 3 μL of a mixture of 0.77% sodium dodecyl sulfate (SDS) and 77.5 mM Na_2_EDTA. Samples were mixed with 2 μL of loading buffer [60% sucrose, 10 mM Tris-HCl (pH 7.9), 0.5% bromophenol blue, and 0.5% xylene cyanol FF] and incubated at 45 °C for 2 min before being loaded onto 1% agarose gels in 100 mM Tris-borate (pH 8.3) and 2 mM EDTA. Gels were stained with 1 μg/mL ethidium bromide for 30 min. DNA bands were visualized with medium-range ultraviolet light and quantified by scanning densitometry using a Protein Simple AlphaImager HP (HP-Al) digital imaging system. DNA supercoiling was monitored by the conversion of the relaxed to supercoiled plasmid. IC_50_ values represent the concentration of gepotidacin or ciprofloxacin that decreased the supercoiling activity by 50% and were calculated using the analysis “[inhibitor] versus response – variable slope (four parameters) nonlinear least-squares fit” provided by GraphPad Prism 9 using the equation:
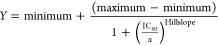
where *Y* = the band intensity for the particular reaction, and *X* = the concentration of the compound.

#### Topoisomerase IV-Catalyzed DNA Decatenation

DNA decatenation assays were based on the procedure of Aldred et al.^44^ Assays contained 1 nM *E. coli* WT topoisomerase IV (1:1 ParC:ParE ratio), 5 nM kDNA, and 1.5 mM ATP in 20 μL of 40 mM HEPES (pH 7.6), 100 mM KGlu, 10 mM Mg(OAc)_2_, and 25 mM NaCl. Assays with the mutant enzymes contained 1 nM ParC^S80L^ or ParC^D79G^. The stated enzyme concentrations reflect those of the holoenzyme (A_2_B_2_). Reactions were carried out in the absence of the compound or in the presence of 0–25 μM gepotidacin or ciprofloxacin, and mixtures were incubated at 37 °C for 20 min (WT), 15 min (ParC^S80L^), or 55 min (ParC^D79G^), which represents the minimum time required to decatenate 80–90% of the DNA in the absence of drug. Reactions were stopped by the addition of 3 μL of a mixture of 0.77% SDS and 77.5 mM Na_2_EDTA. Samples were mixed with 2 μL of loading buffer and incubated at 45 °C for 2 min before being loaded onto 1% agarose gels in 100 mM Tris-borate (pH 8.3) and 2 mM EDTA. Gels were stained with 1 μg/mL ethidium bromide for 30 min. DNA bands were visualized as described above and monitored by the conversion of catenated kDNA into decatenated monomers. IC_50_ values were calculated using the analysis “[inhibitor] versus response – variable slope (four parameters) nonlinear least-squares fit” provided by GraphPad Prism 9 and the equation as described above.

#### DNA Cleavage

DNA cleavage assays with *E. coli* gyrase and topoisomerase IV were based on the procedure of Aldred et al.^100^ Reactions with gyrase were carried out in the absence of compound or in the presence of 0–25 μM gepotidacin or 0–100 μM ciprofloxacin. Reactions with topoisomerase IV were carried out in the absence of a compound or in the presence of 0–5 μM gepotidacin or 0–25 μM ciprofloxacin. Reaction mixtures contained 100 nM WT or mutant (GyrA^S83L^ or GyrA^P35L^) gyrase (1:1 GyrA:GyrB ratio), or 20 nM WT or mutant (ParC^S80L^ or ParC^D79G^) topoisomerase IV (1:1 ParC:ParE ratio). Stated enzyme concentrations reflect those of the holoenzyme (A_2_B_2_). Assay mixtures contained 10 nM negatively supercoiled pBR322 in a total volume of 20 μL of cleavage buffer [40 mM Tris-HCl (pH 7.9), 50 mM NaCl, 10 mM MgCl_2_, and 12.5% glycerol]. Some reactions contained 1.5 mM ATP. Samples were incubated at 37 °C for 20 min with gyrase and for 10 min with topoisomerase IV. Enzyme–DNA cleavage complexes were trapped by adding 2 μL of 5% SDS, followed by 2 μL of 250 mM Na_2_EDTA. Proteinase K (Sigma-Aldrich) (2 μL of 0.8 mg/mL) was added, and mixtures were incubated at 45 °C for 30 min to digest the enzyme. Samples were mixed with 2 μL of loading buffer and incubated at 45 °C for 2 min before being loaded onto 1% agarose gels. Reaction products were subjected to electrophoresis in 40 mM Tris-acetate (pH 8.3) and 2 mM EDTA containing 0.5 μg/mL ethidium bromide. DNA bands were visualized and quantified, as described above. DNA single- or double-stranded cleavage was monitored by the conversion of supercoiled plasmid to nicked or linear molecules, respectively, and quantified by comparison to a control reaction in which an equal amount of DNA was digested with restriction endonuclease *Eco*RI (New England BioLabs). CC_50_ values represent the concentration of gepotidacin or ciprofloxacin that induced 50% of maximal DNA cleavage and were calculated using the analysis “[agonist] versus response – variable slope (four parameters) nonlinear least-squares fit” provided by GraphPad Prism 9 using the equation:
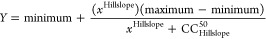
where *Y* = the band intensity for the particular reaction and *X* = the concentration of the compound.

For assays that monitored competition between gepotidacin and ciprofloxacin, the level of double-stranded DNA cleavage induced by 50 μM (gyrase) or 10 μM (topoisomerase IV) ciprofloxacin in the absence of gepotidacin was set to 100%. As increasing concentrations of gepotidacin were added (0–100 or 0–25 μM, respectively), the decrease in double-stranded and increase in single-stranded breaks were quantified. The maximal levels of single-stranded DNA cleavage were set to 100%. In these experiments, competition IC_50_ values were calculated using the analysis “[inhibitor] vs response:variable slope (four parameters) nonlinear least-squares fit” provided by GraphPad Prism 9 described above in the section on gyrase-catalyzed DNA supercoiling.

#### Persistence of DNA Cleavage Complexes

Persistence of cleavage complexes was monitored in the absence or presence of gepotidacin or ciprofloxacin using the procedure of Aldred et al.^44^ Initial gyrase reaction mixtures contained 500 nM WT gyrase, 50 nM negatively supercoiled pBR322, and 10 μM gepotidacin or 50 μM ciprofloxacin in 20 μL of cleavage buffer. CaCl_2_ replaced MgCl_2_ to raise baseline levels of cleavage in experiments without compound. Initial topoisomerase IV reaction mixtures contained 100 nM WT topoisomerase IV, 50 nM negatively supercoiled pBR322, and 0.1 μM gepotidacin or 10 μM ciprofloxacin in 20 μL of cleavage buffer. Topoisomerase IV has high basal activity; therefore, CaCl_2_ was not used in experiments without compounds. Cleavage complexes were allowed to form at 37 °C for 20 min with gyrase and 10 min with topoisomerase IV, then diluted 20-fold in divalent cation-free cleavage buffer. Samples were removed at time points from 0 to 120 min, and reactions were stopped, processed, and quantified as described above. Levels of gepotidacin-induced single-stranded breaks or ciprofloxacin-induced double-stranded breaks were set to 100% at time zero, as were double-stranded breaks in the absence of compound. Persistence was monitored as a loss of single- or double-stranded scission over time.

## References

[ref1] Global burden of bacterial antimicrobial resistance in 2019: a systematic analysis. Lancet 2022, 399 (10325), 629–655. 10.1016/S0140-6736(21)02724-0. 35065702 PMC8841637

[ref2] O’Neill J. Antimicrobial resistance: tackling a crisis for the health and wealth of nations; London, 2014.

[ref3] World Health Organization Advisory Group on Integrated Surveillance of Antimicrobial Resistance. Critically important antimicrobials for human medicine: ranking of medically important antimicrobials for risk management of antimicrobial resistance due to non-human use; 2018. https://apps.who.int/iris/bitstream/handle/10665/312266/9789241515528-eng.pdf (accessed Jan 31, 2024).

[ref4] Drlica K.; Hiasa H.; Kerns R.; Malik M.; Mustaev A.; Zhao X. Quinolones: action and resistance updated. Curr. Top. Med. Chem. 2009, 9 (11), 981–998. 10.2174/156802609789630947. 19747119 PMC3182077

[ref5] Davies J.; Davies D. Origins and evolution of antibiotic resistance. Microbiol. Mol. Biol. Rev. 2010, 74 (3), 417–433. 10.1128/MMBR.00016-10. 20805405 PMC2937522

[ref6] Aldred K. J.; Kerns R. J.; Osheroff N. Mechanism of quinolone action and resistance. Biochemistry 2014, 53 (10), 1565–1574. 10.1021/bi5000564. 24576155 PMC3985860

[ref7] Hooper D. C.; Jacoby G. A. Mechanisms of drug resistance: quinolone resistance. Ann. N.Y. Acad. Sci. 2015, 1354, 12–31. 10.1111/nyas.12830. 26190223 PMC4626314

[ref8] Basarab G. S. Four ways to skin a cat: inhibition of bacterial topoisomerases leading to the clinic. In Antibacterials; Fisher J. F.; Mobashery S.; Miller M. J., Eds.; Topics in Medicinal Chemistry; Springer: Cham, Switzerland, 2017; Vol. 25.

[ref9] Gibson E. G.; Ashley R. E.; Kerns R. J.; Osheroff N. Fluoroquinolone interactions with bacterial type II topoisomerases and target-mediated drug resistance. In Antimicrobial Resistance and Implications for the 21st Century; Drlica K.; Shlaes D.; Fong I. W., Eds.; Springer: Cham, Switzerland, 2018; pp 507–529.

[ref10] Bush N. G.; Diez-Santos I.; Abbott L. R.; Maxwell A. Quinolones: mechanism, lethality and their contributions to antibiotic resistance. Molecules 2020, 25 (23), 5662 10.3390/molecules25235662. 33271787 PMC7730664

[ref11] Bax B. D.; Murshudov G.; Maxwell A.; Germe T. DNA topoisomerase inhibitors: trapping a DNA-cleaving machine in motion. J. Mol. Biol. 2019, 431, 3427–3449. 10.1016/j.jmb.2019.07.008. 31301408 PMC6723622

[ref12] A multi-center, randomized, open-label, non inferiority trial to evaluate the efficacy and safety of a single, oral dose of zoliflodacin compared to a combination of a single intramuscular dose of ceftriaxone and a single oral dose of azithromycin in the treatment of patients with uncomplicated gonorrhoea; ClinTrials.gov, 2021 (accessed Jan 31, 2024).

[ref13] Kokot M.; Anderluh M.; Hrast M.; Minovski N. The structural features of novel bacterial topoisomerase inhibitors that define their activity on topoisomerase IV. J. Med. Chem. 2022, 65 (9), 6431–6440. 10.1021/acs.jmedchem.2c00039. 35503563 PMC9109137

[ref14] Morgan H.; Lipka-Lloyd M.; Warren A. J.; Hughes N.; Holmes J.; Burton N. P.; Mahenthiralingam E.; Bax B. D. A 2.8 A structure of zoliflodacin in a DNA cleavage complex with *Staphylococcus aureus* DNA gyrase. Int. J. Mol. Sci. 2023, 24 (2), 1634 10.3390/ijms24021634. 36675148 PMC9865888

[ref15] GlaxoSmithKline. A phase III, randomized, multicenter, parallel-group, double-blind, double-dummy study in adolescent and adult female participants comparing the efficacy and safety of gepotidacin to nitrofurantoin in the treatment of uncomplicated urinary tract infection (acute cystitis); ClinTrials.gov, 2021 (accessed Jan 31, 2024).

[ref16] GlaxoSmithKline. A phase III, randomized, multicenter, open-label study in adolescent and adult participants comparing the efficacy and safety of gepotidacin to ceftriaxone plus azithromycin in the treatment of uncomplicated urogenital gonorrhea caused by Neisseria gonorrhoeae; ClinTrials.gov, 2022, (accessed Jan 31, 2024).

[ref17] Hackel M. A.; Karlowsky J. A.; Canino M. A.; Sahm D. F.; Scangarella-Oman N. E. In vitro activity of gepotidacin against gram-negative and gram-positive anaerobes. Antimicrob. Agents Chemother. 2022, 66 (2), e0216521 10.1128/aac.02165-21. 34930028 PMC8846401

[ref18] Perry C.; Hossain M.; Powell M.; Raychaudhuri A.; Scangarella-Oman N.; Tiffany C.; Xu S.; Dumont E.; Janmohamed S. Design of two phase III, randomized, multicenter studies comparing gepotidacin with nitrofurantoin for the treatment of uncomplicated urinary tract infection in female participants. Infect. Dis. Ther. 2022, 11 (6), 2297–2310. 10.1007/s40121-022-00706-9. 36271314 PMC9589544

[ref19] Scangarella-Oman N. E.; Hossain M.; Hoover J. L.; Perry C. R.; Tiffany C.; Barth A.; Dumont E. F. Dose selection for phase III clinical evaluation of gepotidacin (GSK2140944) in the treatment of uncomplicated urinary tract infections. Antimicrob. Agents Chemother. 2022, 66 (3), e0149221 10.1128/aac.01492-21. 34978887 PMC8923173

[ref20] Arends S. J. R.; Butler D.; Scangarella-Oman N.; Castanheira M.; Mendes R. E. Antimicrobial activity of gepotidacin tested against *Escherichia coli* and *Staphylococcus saprophyticus* isolates causing urinary tract infections in medical centers worldwide (2019 to 2020). Antimicrob. Agents Chemother. 2023, 67, e0152522 10.1128/aac.01525-22. 36877017 PMC10112209

[ref21] Scangarella-Oman N. E.; Hossain M.; Perry C. R.; Tiffany C.; Powell M.; Swift B.; Dumont E. F. Dose selection for a phase III study evaluating gepotidacin (GSK2140944) in the treatment of uncomplicated urogenital gonorrhoea. Sex. Transm. Infect. 2023, 99 (1), 64–69. 10.1136/sextrans-2022-055518. 36411033 PMC9887395

[ref22] Watkins R. R.; Thapaliya D.; Lemonovich T. L.; Bonomo R. A. Gepotidacin: a novel, oral, ‘first-in-class’ triazaacenaphthylene antibiotic for the treatment of uncomplicated urinary tract infections and urogenital gonorrhoea. J. Antimicrob. Chemother. 2023, 78, 1137 10.1093/jac/dkad060. 36883591

[ref23] GlaxoSmithKline. EAGLE-2 and EAGLE-3 phase III trials for gepotidacin stopped early for efficacy following pre-planned interim analysis by Independent Data Monitoring Committee; 2022 (accessed Jan 31, 2024).

[ref24] GlaxoSmithKline. Gepotidacin’s positive phase III data shows potential to be the first in a new class of oral antibiotics for uncomplicated urinary tract infections in over 20 years; 2023 (accessed Jan 31, 2024).

[ref25] Deweese J. E.; Osheroff M. A.; Osheroff N. DNA topology and topoisomerases: teaching a ″knotty″ subject. Biochem. Mol. Biol. Educ. 2009, 37 (1), 2–10. 10.1002/bmb.20244. PMC2643378 19225573

[ref26] Liu Z.; Deibler R. W.; Chan H. S.; Zechiedrich L. The why and how of DNA unlinking. Nucleic Acids Res. 2009, 37 (3), 661–671. 10.1093/nar/gkp041. 19240147 PMC2647305

[ref27] Bush N. G.; Evans-Roberts K.; Maxwell A. DNA topoisomerases. EcoSal Plus 2015, 6, 2 10.1128/ecosalplus.esp-0010-2014. PMC11575854 26435256

[ref28] Ashley R. E.; Osheroff N. Regulation of DNA topology by topoisomerases: mathematics at the molecular level. In Knots, Low-Dimensional Topology and Applications; Adams C. C.; Gordon C. M.; Jones V. F. R.; Kauffman L. H.; Lambropoulou S.; Millett K. C.; Przytycki J. H.; Ricca R.; Sazadanovic R., Eds.; Springer: Cham, Switzerland, 2019; pp 411−433.

[ref29] Dalvie E. D.; Osheroff N. DNA topoisomerases: type II. In The Encyclopedia of Biological Chemistry, 3rd ed.; Allewell N. M., Ed.; Elsevier: Amsterdam, The Netherlands, 2021; Vol. 3, pp 479–486.

[ref30] McKie S. J.; Neuman K. C.; Maxwell A. DNA topoisomerases: advances in understanding of cellular roles and multi-protein complexes via structure-function analysis. Bioessays 2021, 43 (4), e2000286 10.1002/bies.202000286. 33480441 PMC7614492

[ref31] Liu L. F.; Wang J. C. DNA-DNA gyrase complex: the wrapping of the DNA duplex outside the enzyme. Cell 1978, 15 (3), 979–984. 10.1016/0092-8674(78)90281-7. 153201

[ref32] Kampranis S. C.; Maxwell A. Conversion of DNA gyrase into a conventional type II topoisomerase. Proc. Nat. Acad. Sci. U. S. A. 1996, 93 (25), 14416–14421. 10.1073/pnas.93.25.14416. PMC26147 8962066

[ref33] Ullsperger C.; Cozzarelli N. R. Contrasting enzymatic acitivites of topoisomerase IV and DNA gyrase from *Escherichia coli*. J. Biol. Chem. 1996, 271, 31549–31555. 10.1074/jbc.271.49.31549. 8940171

[ref34] Kramlinger V. M.; Hiasa H. The ″GyrA-box″ is required for the ability of DNA gyrase to wrap DNA and catalyze the supercoiling reaction. J. Biol. Chem. 2006, 281 (6), 3738–3742. 10.1074/jbc.M511160200. 16332690

[ref35] Sissi C.; Palumbo M. In front of and behind the replication fork: bacterial type IIA topoisomerases. Cell. Mol. Life Sci. 2010, 67 (12), 2001–2024. 10.1007/s00018-010-0299-5. 20165898 PMC11115839

[ref36] Hirsch J.; Klostermeier D. What makes a type IIA topoisomerase a gyrase or a topo IV?. Nucleic Acids Res. 2021, 49 (11), 6027–6042. 10.1093/nar/gkab270. 33905522 PMC8216471

[ref37] Vos S. M.; Tretter E. M.; Schmidt B. H.; Berger J. M. All tangled up: how cells direct, manage and exploit topoisomerase function. Nat. Rev. Mol. Cell. Biol. 2011, 12 (12), 827–841. 10.1038/nrm3228. 22108601 PMC4351964

[ref38] Kreuzer K. N.; Cozzarelli N. R. *Escherichia coli* mutants thermosensitive for deoxyribonucleic acid gyrase subunit A: effects on deoxyribonucleic acid replication, transcription, and bacteriophage growth. J. Bacteriol. 1979, 140 (2), 424–435. 10.1128/jb.140.2.424-435.1979. 227840 PMC216666

[ref39] Cheng G.; Hao H.; Dai M.; Liu Z.; Yuan Z. Antibacterial action of quinolones: from target to network. Eur. J. Med. Chem. 2013, 66, 555–562. 10.1016/j.ejmech.2013.01.057. 23528390

[ref40] Govender P.; Muller R.; Singh K.; Reddy V.; Eyermann C. J.; Fienberg S.; Ghorpade S. R.; Koekemoer L.; Myrick A.; Schnappinger D.; Engelhart C.; Byl J. A. W.; Osheroff N.; Singh V.; Chibale K.; Basarab G. S. Spiropyrimidinetrione DNA gyrase inhibitors with potent and selective antituberculosis activity. J. Med. Chem. 2022, 65 (9), 6903–6925. 10.1021/acs.jmedchem.2c00266. 35500229 PMC9233935

[ref41] Byl J. A. W.; Mueller R.; Bax B.; Basarab G. S.; Chibale K.; Osheroff N. A series of spiropyrimidinetriones that enhances DNA cleavage mediated by *Mycobacterium tuberculosis* gyrase. ACS Infect. Dis. 2023, 9 (3), 706–715. 10.1021/acsinfecdis.3c00012. 36802491 PMC10006343

[ref42] Bax B. D.; Chan P. F.; Eggleston D. S.; Fosberry A.; Gentry D. R.; Gorrec F.; Giordano I.; Hann M. M.; Hennessy A.; Hibbs M.; Huang J.; Jones E.; Jones J.; Brown K. K.; Lewis C. J.; May E. W.; Saunders M. R.; Singh O.; Spitzfaden C. E.; Shen C.; Shillings A.; Theobald A. J.; Wohlkonig A.; Pearson N. D.; Gwynn M. N. Type IIA topoisomerase inhibition by a new class of antibacterial agents. Nature 2010, 466 (7309), 935–940. 10.1038/nature09197. 20686482

[ref43] Wohlkonig A.; Chan P. F.; Fosberry A. P.; Homes P.; Huang J.; Kranz M.; Leydon V. R.; Miles T. J.; Pearson N. D.; Perera R. L.; Shillings A. J.; Gwynn M. N.; Bax B. D. Structural basis of quinolone inhibition of type IIA topoisomerases and target-mediated resistance. Nat. Struct. Mol. Biol. 2010, 17 (9), 1152–1153. 10.1038/nsmb.1892. 20802486

[ref44] Aldred K. J.; McPherson S. A.; Wang P.; Kerns R. J.; Graves D. E.; Turnbough C. L.; Osheroff N. Drug interactions with *Bacillus anthracis* topoisomerase IV: biochemical basis for quinolone action and resistance. Biochemistry 2012, 51 (1), 370–381. 10.1021/bi2013905. 22126453 PMC3261753

[ref45] Blower T. R.; Williamson B. H.; Kerns R. J.; Berger J. M. Crystal structure and stability of gyrase-fluoroquinolone cleaved complexes from *Mycobacterium tuberculosis*. Proc. Natl. Acad. Sci. U.S.A. 2016, 113 (7), 1706–1713. 10.1073/pnas.1525047113. 26792525 PMC4763791

[ref46] Heisig P.; Schedletzky H.; Falkenstein-Paul H. Mutations in the gyrA gene of a highly fluoroquinolone-resistant clinical isolate of *Escherichia coli*. Antimicrob. Agents Chemother. 1993, 37 (4), 696–701. 10.1128/AAC.37.4.696. 8388197 PMC187737

[ref47] Khodursky A. B.; Zechiedrich E. L.; Cozzarelli N. R. Topoisomerase IV is a target of quinolones in *Escherichia coli*. Proc. Nat. Acad. Sci. U. S. A. 1995, 92, 11801–11805. 10.1073/pnas.92.25.11801. PMC40490 8524852

[ref48] Morgan-Linnell S. K.; Becnel Boyd L.; Steffen D.; Zechiedrich L. Mechanisms accounting for fluoroquinolone resistance in *Escherichia coli* clinical isolates. Antimicrob. Agents Chemother. 2009, 53 (1), 235–241. 10.1128/AAC.00665-08. 18838592 PMC2612180

[ref49] Aldred K. J.; McPherson S. A.; Turnbough C. L.; Kerns R. J.; Osheroff N. Topoisomerase IV-quinolone interactions are mediated through a water-metal ion bridge: mechanistic basis of quinolone resistance. Nucleic Acids Res. 2013, 41 (8), 4628–4639. 10.1093/nar/gkt124. 23460203 PMC3632122

[ref50] Aldred K. J.; Breland E. J.; McPherson S. A.; Turnbough C. L. Jr.; Kerns R. J.; Osheroff N. *Bacillus anthracis* GrlAV96A topoisomerase IV, a quinolone resistance mutation that does not affect the water-metal ion bridge. Antimicrob. Agents Chemother. 2014, 58 (12), 7182–7187. 10.1128/AAC.03734-14. 25246407 PMC4249509

[ref51] Black M. T.; Stachyra T.; Platel D.; Girard A. M.; Claudon M.; Bruneau J. M.; Miossec C. Mechanism of action of the antibiotic NXL101, a novel nonfluoroquinolone inhibitor of bacterial type II topoisomerases. Antimicrob. Agents Chemother. 2008, 52 (9), 3339–3349. 10.1128/AAC.00496-08. 18625781 PMC2533460

[ref52] Dougherty T. J.; Nayar A.; Newman J. V.; Hopkins S.; Stone G. G.; Johnstone M.; Shapiro A. B.; Cronin M.; Reck F.; Ehmann D. E. NBTI 5463 is a novel bacterial type II topoisomerase inhibitor with activity against gram-negative bacteria and *in vivo* efficacy. Antimicrob. Agents Chemother. 2014, 58 (5), 2657–2664. 10.1128/AAC.02778-13. 24566174 PMC3993248

[ref53] Gibson E. G.; Blower T. R.; Cacho M.; Bax B.; Berger J. M.; Osheroff N. Mechanism of action of *Mycobacterium tuberculosis* gyrase inhibitors: a novel class of gyrase poisons. ACS Infect. Dis. 2018, 4 (8), 1211–1222. 10.1021/acsinfecdis.8b00035. 29746087 PMC6309371

[ref54] Gibson E. G.; Bax B.; Chan P. F.; Osheroff N. Mechanistic and structural basis for the actions of the antibacterial gepotidacin against *Staphylococcus aureus* gyrase. ACS Infect. Dis. 2019, 5, 570–581. 10.1021/acsinfecdis.8b00315. 30757898 PMC6461504

[ref55] Gibson E. G.; Oviatt A. A.; Cacho M.; Neuman K. C.; Chan P. F.; Osheroff N. Bimodal Actions of a Naphthyridone/Aminopiperidine-Based Antibacterial That Targets Gyrase and Topoisomerase IV. Biochemistry 2019, 58 (44), 4447–4455. 10.1021/acs.biochem.9b00805. 31617352 PMC7450530

[ref56] Lu Y.; Papa J. L.; Nolan S.; English A.; Seffernick J. T.; Shkolnikov N.; Powell J.; Lindert S.; Wozniak D. J.; Yalowich J.; Mitton-Fry M. J. Dioxane-linked amide derivatives as novel bacterial topoisomerase inhibitors against Gram-positive *Staphylococcus aureus*. ACS Med. Chem. Lett. 2020, 11 (12), 2446–2454. 10.1021/acsmedchemlett.0c00428. 33335666 PMC7734797

[ref57] Lu Y.; Vibhute S.; Li L.; Okumu A.; Ratigan S. C.; Nolan S.; Papa J. L.; Mann C. A.; English A.; Chen A.; Seffernick J. T.; Koci B.; Duncan L. R.; Roth B.; Cummings J. E.; Slayden R. A.; Lindert S.; McElroy C. A.; Wozniak D. J.; Yalowich J.; Mitton-Fry M. J. Optimization of topoIV potency, ADMET properties, and hERG inhibition of 5-amino-1,3-dioxane-linked novel bacterial topoisomerase inhibitors: identification of a lead with in vivo efficacy against MRSA. J. Med. Chem. 2021, 64 (20), 15214–15249. 10.1021/acs.jmedchem.1c01250. 34614347

[ref58] Mitton-Fry M. J.; Brickner S. J.; Hamel J. C.; Brennan L.; Casavant J. M.; Chen M.; Chen T.; Ding X.; Driscoll J.; Hardink J.; Hoang T.; Hua E.; Huband M. D.; Maloney M.; Marfat A.; McCurdy S. P.; McLeod D.; Plotkin M.; Reilly U.; Robinson S.; Schafer J.; Shepard R. M.; Smith J. F.; Stone G. G.; Subramanyam C.; Yoon K.; Yuan W.; Zaniewski R. P.; Zook C. Novel quinoline derivatives as inhibitors of bacterial DNA gyrase and topoisomerase IV. Bioorg. Med. Chem. Lett. 2013, 23 (10), 2955–2961. 10.1016/j.bmcl.2013.03.047. 23566517

[ref59] Surivet J. P.; Zumbrunn C.; Rueedi G.; Hubschwerlen C.; Bur D.; Bruyere T.; Locher H.; Ritz D.; Keck W.; Seiler P.; Kohl C.; Gauvin J. C.; Mirre A.; Kaegi V.; Dos Santos M.; Gaertner M.; Delers J.; Enderlin-Paput M.; Boehme M. Design, synthesis, and characterization of novel tetrahydropyran-based bacterial topoisomerase inhibitors with potent anti-gram-positive activity. J. Med. Chem. 2013, 56 (18), 7396–7415. 10.1021/jm400963y. 23968485

[ref60] Singh S. B.; Kaelin D. E.; Wu J.; Miesel L.; Tan C. M.; Meinke P. T.; Olsen D.; Lagrutta A.; Bradley P.; Lu J.; Patel S.; Rickert K. W.; Smith R. F.; Soisson S.; Wei C.; Fukuda H.; Kishii R.; Takei M.; Fukuda Y. Oxabicyclooctane-linked novel bacterial topoisomerase inhibitors as broad spectrum antibacterial agents. ACS Med. Chem. Lett. 2014, 5 (5), 609–614. 10.1021/ml500069w. 24900889 PMC4027601

[ref61] Surivet J. P.; Zumbrunn C.; Rueedi G.; Bur D.; Bruyere T.; Locher H.; Ritz D.; Seiler P.; Kohl C.; Ertel E. A.; Hess P.; Gauvin J. C.; Mirre A.; Kaegi V.; Dos Santos M.; Kraemer S.; Gaertner M.; Delers J.; Enderlin-Paput M.; Weiss M.; Sube R.; Hadana H.; Keck W.; Hubschwerlen C. Novel tetrahydropyran-based bacterial topoisomerase inhibitors with potent anti-gram positive activity and improved safety profile. J. Med. Chem. 2015, 58 (2), 927–942. 10.1021/jm501590q. 25494934

[ref62] Tan C. M.; Gill C. J.; Wu J.; Toussaint N.; Yin J.; Tsuchiya T.; Garlisi C. G.; Kaelin D.; Meinke P. T.; Miesel L.; Olsen D. B.; Lagrutta A.; Fukuda H.; Kishii R.; Takei M.; Oohata K.; Takeuchi T.; Shibue T.; Takano H.; Nishimura A.; Fukuda Y.; Singh S. B. *In vitro* and *in vivo* characterization of the novel oxabicyclooctane-linked bacterial topoisomerase inhibitor AM-8722, a selective, potent inhibitor of bacterial DNA gyrase. Antimicrob. Agents Chemother. 2016, 60 (8), 4830–4839. 10.1128/AAC.00619-16. 27246784 PMC4958163

[ref63] Surivet J. P.; Zumbrunn C.; Bruyere T.; Bur D.; Kohl C.; Locher H. H.; Seiler P.; Ertel E. A.; Hess P.; Enderlin-Paput M.; Enderlin-Paput S.; Gauvin J. C.; Mirre A.; Hubschwerlen C.; Ritz D.; Rueedi G. Synthesis and characterization of tetrahydropyran-based bacterial topoisomerase inhibitors with antibacterial activity against gram-negative bacteria. J. Med. Chem. 2017, 60 (9), 3776–3794. 10.1021/acs.jmedchem.6b01831. 28406300

[ref64] Charrier C.; Salisbury A. M.; Savage V. J.; Duffy T.; Moyo E.; Chaffer-Malam N.; Ooi N.; Newman R.; Cheung J.; Metzger R.; McGarry D.; Pichowicz M.; Sigerson R.; Cooper I. R.; Nelson G.; Butler H. S.; Craighead M.; Ratcliffe A. J.; Best S. A.; Stokes N. R. Novel bacterial topoisomerase inhibitors with potent broad-spectrum activity against drug-resistant bacteria. Antimicrob. Agents Chemother. 2017, 61 (5), e02100–02116. 10.1128/AAC.02100-16. 28223393 PMC5404544

[ref65] Mitton-Fry M. J.; Brickner S. J.; Hamel J. C.; Barham R.; Brennan L.; Casavant J. M.; Ding X.; Finegan S.; Hardink J.; Hoang T.; Huband M. D.; Maloney M.; Marfat A.; McCurdy S. P.; McLeod D.; Subramanyam C.; Plotkin M.; Reilly U.; Schafer J.; Stone G. G.; Uccello D. P.; Wisialowski T.; Yoon K.; Zaniewski R.; Zook C. Novel 3-fluoro-6-methoxyquinoline derivatives as inhibitors of bacterial DNA gyrase and topoisomerase IV. Bioorg. Med. Chem. Lett. 2017, 27 (15), 3353–3358. 10.1016/j.bmcl.2017.06.009. 28610977

[ref66] Li L.; Okumu A.; Dellos-Nolan S.; Li Z.; Karmahapatra S.; English A.; Yalowich J. C.; Wozniak D. J.; Mitton-Fry M. J. Synthesis and anti-staphylococcal activity of novel bacterial topoisomerase inhibitors with a 5-amino-1,3-dioxane linker moiety. Bioorg. Med. Chem. Lett. 2018, 28 (14), 2477–2480. 10.1016/j.bmcl.2018.06.003. 29871847

[ref67] Li L.; Okumu A. A.; Nolan S.; English A.; Vibhute S.; Lu Y.; Hervert-Thomas K.; Seffernick J. T.; Azap L.; Cole S. L.; Shinabarger D.; Koeth L. M.; Lindert S.; Yalowich J. C.; Wozniak D. J.; Mitton-Fry M. J. 1,3-dioxane-linked bacterial topoisomerase inhibitors with enhanced antibacterial activity and reduced hERG inhibition. ACS Infect. Dis. 2019, 5 (7), 1115–1128. 10.1021/acsinfecdis.8b00375. 31041863

[ref68] Okumu A.; Lu Y.; Dellos-Nolan S.; Papa J. L.; Koci B.; Cockroft N. T.; Gallucci J.; Wozniak D. J.; Yalowich J. C.; Mitton-Fry M. J. Novel bacterial topoisomerase inhibitors derived from isomannide. Eur. J. Med. Chem. 2020, 199, 112324 10.1016/j.ejmech.2020.112324. 32402932

[ref69] Lu Y.; Mann C. A.; Nolan S.; Collins J. A.; Parker E.; Papa J.; Vibhute S.; Jahanbakhsh S.; Thwaites M.; Hufnagel D.; Hazbon M. H.; Moreno J.; Stedman T. T.; Wittum T.; Wozniak D. J.; Osheroff N.; Yalowich J. C.; Mitton-Fry M. J. 1,3-Dioxane-linked novel bacterial topoisomerase inhibitors: expanding structural diversity and the antibacterial spectrum. ACS Med. Chem. Lett. 2022, 13 (6), 955–963. 10.1021/acsmedchemlett.2c00111. 35707162 PMC9189870

[ref70] Miles T. J.; Hennessy A. J.; Bax B.; Brooks G.; Brown B. S.; Brown P.; Cailleau N.; Chen D.; Dabbs S.; Davies D. T.; Esken J. M.; Giordano I.; Hoover J. L.; Huang J.; Jones G. E.; Sukmar S. K.; Spitzfaden C.; Markwell R. E.; Minthorn E. A.; Rittenhouse S.; Gwynn M. N.; Pearson N. D. Novel hydroxyl tricyclics (e.g., GSK966587) as potent inhibitors of bacterial type IIA topoisomerases. Bioorg. Med. Chem. Lett. 2013, 23 (19), 5437–5441. 10.1016/j.bmcl.2013.07.013. 23968823

[ref71] Miles T. J.; Hennessy A. J.; Bax B.; Brooks G.; Brown B. S.; Brown P.; Cailleau N.; Chen D.; Dabbs S.; Davies D. T.; Esken J. M.; Giordano I.; Hoover J. L.; Jones G. E.; Kusalakumari Sukmar S. K.; Markwell R. E.; Minthorn E. A.; Rittenhouse S.; Gwynn M. N.; Pearson N. D. Novel tricyclics (e.g., GSK945237) as potent inhibitors of bacterial type IIA topoisomerases. Bioorg. Med. Chem. Lett. 2016, 26 (10), 2464–2469. 10.1016/j.bmcl.2016.03.106. 27055939

[ref72] Vanden Broeck A.; Lotz C.; Ortiz J.; Lamour V. Cryo-EM structure of the complete *E. coli* DNA gyrase nucleoprotein complex. Nat. Commun. 2019, 10 (1), 4935 10.1038/s41467-019-12914-y. 31666516 PMC6821735

[ref73] Laponogov I.; Pan X. S.; Veselkov D. A.; McAuley K. E.; Fisher L. M.; Sanderson M. R. Structural basis of gate-DNA breakage and resealing by type II topoisomerases. PLoS One 2010, 5 (6), e11338 10.1371/journal.pone.0011338. 20596531 PMC2893164

[ref74] Chan P. F.; Srikannathasan V.; Huang J.; Cui H.; Fosberry A. P.; Gu M.; Hann M. M.; Hibbs M.; Homes P.; Ingraham K.; Pizzollo J.; Shen C.; Shillings A. J.; Spitzfaden C. E.; Tanner R.; Theobald A. J.; Stavenger R. A.; Bax B. D.; Gwynn M. N. Structural basis of DNA gyrase inhibition by antibacterial QPT-1, anticancer drug etoposide and moxifloxacin. Nat. Commun. 2015, 6, 10048 10.1038/ncomms10048. 26640131 PMC4686662

[ref75] Szili P.; Draskovits G.; Revesz T.; Bogar F.; Balogh D.; Martinek T.; Daruka L.; Spohn R.; Vasarhelyi B. M.; Czikkely M.; Kintses B.; Grezal G.; Ferenc G.; Pal C.; Nyerges A. Rapid evolution of reduced susceptibility against a balanced dual-targeting antibiotic through stepping-stone mutations. Antimicrob. Agents Chemother. 2019, 63 (9), 1 10.1128/AAC.00207-19. PMC6709476 31235632

[ref76] Blanche F.; Cameron B.; Bernard F. X.; Maton L.; Manse B.; Ferrero L.; Ratet N.; Lecoq C.; Goniot A.; Bisch D.; Crouzet J. Differential behaviors of *Staphylococcus aureus* and *Escherichia coli* type II DNA topoisomerases. Antimicrob. Agents Chemother. 1996, 40 (12), 2714–2720. 10.1128/AAC.40.12.2714. 9124828 PMC163609

[ref77] Pan X. S.; Fisher L. M. DNA gyrase and topoisomerase IV are dual targets of clinafloxacin action in *Streptococcus pneumoniae*. Antimicrob. Agents Chemother. 1998, 42 (11), 2810–2816. 10.1128/AAC.42.11.2810. 9797208 PMC105948

[ref78] Pan X.-S.; Fisher L. M. *Streptococcus pneumoniae* DNA gyrase and topoisomerase IV: overexpression, purification, and differential inhibition by fluoroquinolones. Antimicrob. Agents Chemother. 1999, 43, 1129–1136. 10.1128/AAC.43.5.1129. 10223925 PMC89122

[ref79] Aedo S.; Tse-Dinh Y.-C. Isolation and quantitation of topoisomerase complexes accumulated on *Escherichia coli* chromosomal DNA. Antimicrob. Agents Chemother. 2012, 56 (11), 5458–5464. 10.1128/AAC.01182-12. 22869559 PMC3486612

[ref80] Nayar A. S.; Dougherty T. J.; Reck F.; Thresher J.; Gao N.; Shapiro A. B.; Ehmann D. E. Target-based resistance in *Pseudomonas aeruginosa* and *Escherichia coli* to NBTI 5463, a novel bacterial type II topoisomerase inhibitor. Antimicrob. Agents Chemother. 2015, 59 (1), 331–337. 10.1128/AAC.04077-14. 25348539 PMC4291369

[ref81] Flamm R. K.; Farrell D. J.; Rhomberg P. R.; Scangarella-Oman N. E.; Sader H. S. Gepotidacin (GSK2140944) in vitro activity against gram-positive and gram-negative bacteria. Antimicrob. Agents Chemother. 2017, 61 (7), e00468-417 10.1128/AAC.00468-17. 28483959 PMC5487655

[ref82] Nyerges A.; Csorgo B.; Draskovits G.; Kintses B.; Szili P.; Ferenc G.; Revesz T.; Ari E.; Nagy I.; Balint B.; Vasarhelyi B. M.; Bihari P.; Szamel M.; Balogh D.; Papp H.; Kalapis D.; Papp B.; Pal C. Directed evolution of multiple genomic loci allows the prediction of antibiotic resistance. Proc. Nat. Acad. Sci. U. S. A. 2018, 115 (25), E5726–E5735. 10.1073/pnas.1801646115. PMC6016788 29871954

[ref83] Lahiri S. D.; Kutschke A.; McCormack K.; Alm R. A. Insights into the mechanism of inhibition of novel bacterial topoisomerase inhibitors from characterization of resistant mutants of *Staphylococcus aureus*. Antimicrob. Agents Chemother. 2015, 59 (9), 5278–5287. 10.1128/AAC.00571-15. 26077256 PMC4538526

[ref84] Ashley R. E.; Lindsey R. H. Jr.; McPherson S. A.; Turnbough C. L. Jr.; Kerns R. J.; Osheroff N. Interactions between quinolones and *Bacillus anthracis* gyrase and the basis of drug resistance. Biochemistry 2017, 56 (32), 4191–4200. 10.1021/acs.biochem.7b00203. 28708938 PMC5560241

[ref85] Barnard F. M.; Maxwell A. Interaction between DNA gyrase and quinolones: effects of alanine mutations at GyrA subunit residues Ser(83) and Asp(87). Antimicrob. Agents Chemother. 2001, 45 (7), 1994–2000. 10.1128/AAC.45.7.1994-2000.2001. 11408214 PMC90591

[ref86] Deweese J. E.; Osheroff N. The use of divalent metal ions by type II topoisomerases. Metallomics 2010, 2 (7), 450–459. 10.1039/c003759a. 20703329 PMC2918885

[ref87] Pitts S. L.; Liou G. F.; Mitchenall L. A.; Burgin A. B.; Maxwell A.; Neuman K. C.; Osheroff N. Use of divalent metal ions in the DNA cleavage reaction of topoisomerase IV. Nucleic Acids Res. 2011, 39 (11), 4808–4817. 10.1093/nar/gkr018. 21300644 PMC3113566

[ref88] Gellert M.; Mizuuchi K.; O’Dea M. H.; Nash H. A. DNA gyrase: an enzyme that introduces superhelical turns into DNA. Proc. Nat. Acad. Sci. U. S. A. 1976, 73, 3872–3876. 10.1073/pnas.73.11.3872. PMC431247 186775

[ref89] Kato J.; Suzuki H.; Ikeda H. Purification and characterization of DNA topoisomerase IV in *Escherichia coli*. J. Biol. Chem. 1992, 267 (36), 25676–25684. 10.1016/S0021-9258(18)35660-6. 1334483

[ref90] Greiner J. V.; Glonek T. Intracellular ATP concentration and implication for cellular evolution. Biology 2021, 10 (11), 1166 10.3390/biology10111166. 34827159 PMC8615055

[ref91] Bandele O. J.; Osheroff N. The efficacy of topoisomerase II-targeted anticancer agents reflects the persistence of drug-induced cleavage complexes in cells. Biochemistry 2008, 47 (45), 11900–11908. 10.1021/bi800981j. 18922022 PMC2626429

[ref92] Gentry A. C.; Pitts S. L.; Jablonsky M. J.; Bailly C.; Graves D. E.; Osheroff N. Interactions between the etoposide derivative F14512 and human type II topoisomerases: implications for the C4 spermine moiety in promoting enzyme-mediated DNA cleavage. Biochemistry 2011, 50 (15), 3240–3249. 10.1021/bi200094z. 21413765 PMC3086367

[ref93] Jian J. Y.; McCarty K. D.; Byl J. A. W.; Guengerich F. P.; Neuman K. C.; Osheroff N. Basis for the discrimination of supercoil handedness during DNA cleavage by human and bacterial type II topoisomerases. Nucleic Acids Res. 2023, 51 (8), 3888–3902. 10.1093/nar/gkad190. 36999602 PMC10164583

[ref94] Papillon J.; Menetret J. F.; Batisse C.; Helye R.; Schultz P.; Potier N.; Lamour V. Structural insight into negative DNA supercoiling by DNA gyrase, a bacterial type 2A DNA topoisomerase. Nucleic Acids Res. 2013, 41 (16), 7815–7827. 10.1093/nar/gkt560. 23804759 PMC3763546

[ref95] Willmott C. J.; Maxwell A. A single point mutation in the DNA gyrase A protein greatly reduces binding of fluoroquinolones to the gyrase-DNA complex. Antimicrob. Agents Chemother. 1993, 37 (1), 126–127. 10.1128/AAC.37.1.126. 8381633 PMC187618

[ref96] Gruger T.; Nitiss J. L.; Maxwell A.; Zechiedrich E. L.; Heisig P.; Seeber S.; Pommier Y.; Strumberg D. A mutation in *Escherichia coli* DNA gyrase conferring quinolone resistance results in sensitivity to drugs targeting eukaryotic topoisomerase II. Antimicrob. Agents Chemother. 2004, 48 (12), 4495–4504. 10.1128/AAC.48.12.4495-4504.2004. 15561817 PMC529191

[ref97] Oppegard L. M.; Streck K. R.; Rosen J. D.; Schwanz H. A.; Drlica K.; Kerns R. J.; Hiasa H. Comparison of in vitro activities of fluoroquinolone-like 2,4- and 1,3-diones. Antimicrob. Agents Chemother. 2010, 54 (7), 3011–3014. 10.1128/AAC.00190-10. 20404126 PMC2897325

[ref98] Germe T.; Voros J.; Jeannot F.; Taillier T.; Stavenger R. A.; Bacque E.; Maxwell A.; Bax B. D. A new class of antibacterials, the imidazopyrazinones, reveal structural transitions involved in DNA gyrase poisoning and mechanisms of resistance. Nucleic Acids Res. 2018, 46 (8), 4325 10.1093/nar/gky241. 29596599 PMC5934632

[ref99] Carter H. E.; Wildman B.; Schwanz H. A.; Kerns R. J.; Aldred K. J. Role of the water-metal ion bridge in quinolone interactions with *Escherichia coli* gyrase. Int. J. Mol. Sci. 2023, 24 (3), 2879 10.3390/ijms24032879. 36769202 PMC9917921

[ref100] Aldred K. J.; Breland E. J.; Vlčková V.; Strub M. P.; Neuman K. C.; Kerns R. J.; Osheroff N. Role of the water-metal ion bridge in mediating interactions between quinolones and *Escherichia coli* topoisomerase IV. Biochemistry 2014, 53 (34), 5558–5567. 10.1021/bi500682e. 25115926 PMC4151693

[ref101] Fiorani P.; Bjornsti M. A. Mechanisms of DNA topoisomerase I-induced cell killing in the yeast *Saccharomyces cerevisiae*. Ann. N.Y. Acad. Sci. 2000, 922, 65–75. 10.1111/j.1749-6632.2000.tb07026.x. 11193926

[ref102] Muslimovic A.; Nystrom S.; Gao Y.; Hammarsten O. Numerical analysis of etoposide induced DNA breaks. PLoS One 2009, 4 (6), e5859 10.1371/journal.pone.0005859. 19516899 PMC2689654

[ref103] Pommier Y. Drugging topoisomerases: lessons and challenges. ACS Chem. Biol. 2013, 8 (1), 82–95. 10.1021/cb300648v. 23259582 PMC3549721

[ref104] Thomas A.; Pommier Y. Targeting topoisomerase I in the era of precision medicine. Clin. Cancer Res. 2019, 25 (22), 6581–6589. 10.1158/1078-0432.CCR-19-1089. 31227499 PMC6858945

[ref105] Cheng B.; Shukla S.; Vasunilashorn S.; Mukhopadhyay S.; Tse-Dinh Y. C. Bacterial cell killing mediated by topoisomerase I DNA cleavage activity. J. Biol. Chem. 2005, 280 (46), 38489–38495. 10.1074/jbc.M509722200. 16159875 PMC1351368

[ref106] Datsenko K. A.; Wanner B. L. One-step inactivation of chromosomal genes in *Escherichia coli* K-12 using PCR products. Proc. Nat. Acad. Sci. U. S. A. 2000, 97 (12), 6640–6645. 10.1073/pnas.120163297. PMC18686 10829079

[ref107] Englund P. T. The replication of kinetoplast DNA networks in *Crithidia fasciculata*. Cell 1978, 14 (1), 157–168. 10.1016/0092-8674(78)90310-0. 667931

[ref108] Dong S.; McPherson S. A.; Wang Y.; Li M.; Wang P.; Turnbough C. L.; Pritchard D. G. Characterization of the enzymes encoded by the anthrose biosynthetic operon of *Bacillus anthracis*. J. Bacteriol. 2010, 192 (19), 5053–5062. 10.1128/JB.00568-10. 20675481 PMC2944512

[ref109] Peng H.; Marians K. J. *Escherichia coli* topoisomerase IV. Purification, characterization, subunit structure, and subunit interactions. J. Biol. Chem. 1993, 268 (32), 24481–24490. 10.1016/S0021-9258(20)80551-1. 8227000

[ref110] Corbett K. D.; Schoeffler A. J.; Thomsen N. D.; Berger J. M. The structural basis for substrate specificity in DNA topoisomerase IV. J. Mol. Biol. 2005, 351 (3), 545–561. 10.1016/j.jmb.2005.06.029. 16023670

[ref111] CLSI. Methods for dilution antimicrobial susceptibility tests for bacteria that grow aerobically: approved standard; Clinical and Laboratory Standards Institute: Wayne, PA, 2009.

[ref112] Aldred K. J.; Blower T. R.; Kerns R. J.; Berger J. M.; Osheroff N. Fluoroquinolone interactions with *Mycobacterium tuberculosis* gyrase: enhancing drug activity against wild-type and resistant gyrase. Proc. Nat. Acad. Sci. U. S. A. 2016, 113 (7), E839–E846. 10.1073/pnas.1525055113. PMC4763725 26792518

[ref113] Wagenlehner F.; Perry C. R; Hooton T. M; Scangarella-Oman N. E; Millns H.; Powell M.; Jarvis E.; Dennison J.; Sheets A.; Butler D.; Breton J.; Janmohamed S. Oral gepotidacin versus nitrofurantoin in patients with uncomplicated urinary tract infection (EAGLE-2 and EAGLE-3): two randomised, controlled, double-blind, double-dummy, phase 3, non-inferiority trials. Lancet 2024, 403 (10428), 741–755. 10.1016/S0140-6736(23)02196-7. 38342126

